# Structure-based discovery of positive allosteric modulators of the A_1_ adenosine receptor

**DOI:** 10.1073/pnas.2421687122

**Published:** 2025-07-07

**Authors:** Anh T. N. Nguyen, Nicolas Panel, Duc Duy Vo, Bui San Thai, Ling Yeong Chia, Cam Sinh Lu, Shane D. Hellyer, Monica Langiu, Manuela Jörg, Karen J. Gregory, Jan Kihlberg, Paul J. White, Peter J. Scammells, Arthur Christopoulos, Jens Carlsson, Lauren T. May

**Affiliations:** ^a^Drug Discovery Biology Theme, Monash Institute of Pharmaceutical Sciences, Monash University, Parkville, VIC 3052, Australia; ^b^Science for Life Laboratory, Department of Cell and Molecular Biology, Uppsala University, Uppsala SE-751 24, Sweden; ^c^Department of Chemistry-Biomedical Centre (BMC), Uppsala University, Uppsala SE-75123, Sweden; ^d^Medicinal Chemistry Theme, Monash Institute of Pharmaceutical Sciences, Monash University, Parkville, VIC 3052, Australia; ^e^Australian Research Council Centre for Cryo-electron Microscopy of Membrane Proteins, Monash Institute of Pharmaceutical Sciences, Monash University, Parkville, VIC 3052, Australia

**Keywords:** G protein–coupled receptor, A_1_ adenosine receptor, drug discovery, structure-based virtual screening, allosteric modulation

## Abstract

Allosteric modulation of G protein–coupled receptors (GPCRs) is an exciting strategy for developing new therapeutic agents, and it has several advantages over more commonly used orthosteric drugs. Recently determined GPCR structures have revealed allosteric pockets facing the lipid bilayer, enabling rational drug design. Here, we develop a virtual screening strategy to discover ligands of extrahelical binding pockets and apply this approach to the adenosine A_1_ receptor (A_1_R). The A_1_R is a high-value therapeutic target for ischemia–reperfusion injury and chronic neuropathic pain. Developing effective A_1_R therapeutics remains challenging due to high structural conservation across orthosteric binding sites and on-target unwanted effects stimulated by prototypical A_1_R agonists, such as bradycardia and atrioventricular block. However, A_1_R positive allosteric modulators (PAMs) acting through spatially distinct allosteric sites can fine-tune A_1_R activity with high subtype selectivity and spatiotemporal specificity, thereby overcoming current limitations. A chemical library of 160 million compounds was computationally docked to the allosteric pocket identified in a cryo-EM structure of the A_1_R, and a set of 26 top-ranked compounds were selected for experimental evaluation. Pharmacological evaluation of these, and structure-guided hit optimization, led to the discovery of subtype-selective A_1_R PAMs. These compounds demonstrated minimal allosteric agonism and negligible impact on A_1_R-mediated beat rate of an orthosteric agonist. The discovered PAMs pave the way for potential treatments for neuropathic pain and ischemia–reperfusion injury without accompanying side effects. Our results demonstrate the utility of a synergistic computational and experimental approach in GPCR drug discovery.

## Significance

Our research marks a pivotal advance in allosteric drug development. Insights into G protein–coupled receptor (GPCR) extrahelical allosteric binding pockets necessitated the development of virtual screening techniques that account for membrane interaction. Based on molecular dynamics simulations, our refined computational approach enhanced screening performance for such pockets in multiple GPCRs, offering a template for other structurally defined GPCRs. At the A_1_ receptor (A_1_R), the PAMs **54** and **56** modulated neuronal A_1_R activity without unwanted cardiac effects, a common hindrance in adenosine receptor therapeutics. Our research showcases the efficacy of blending experimental GPCR structures with computational techniques and paves the way for the broader application of this strategy in allosteric drug development.

The G protein–coupled receptor (GPCR) superfamily of transmembrane-spanning proteins is integral for cellular communication in health and disease. At least 34% of current FDA-approved drugs target GPCRs (1, 2). The vast majority of GPCR medicines mediate their effect by engaging the primary, orthosteric site recognized by the endogenous agonist. However, many GPCRs remain challenging targets for drug discovery due to the lack of subtype-selective orthosteric agonists. Developing allosteric ligands that modulate GPCR activity through interacting with a spatially distinct binding site provides the opportunity to develop new therapeutics with enhanced subtype selectivity and spatiotemporal specificity (3). Positive allosteric modulators (PAMs) or negative allosteric modulators (NAMs) enhance or inhibit orthosteric ligand binding and/or function, respectively (3), quantified by cooperativity factors. Allosteric ligand affinity, efficacy, and cooperativity can be tailored to achieve an optimal therapeutic profile (4). Allosteric ligands also have the potential to impart biased signaling (5), stabilizing distinct GPCR conformations and thereby conferring pathway-specific effects.

The adenosine family of GPCRs, particularly the widely expressed A_1_ adenosine receptor (A_1_R), are high-value therapeutic targets for conditions such as neuropathic pain and ischemia–reperfusion injury (6). Effective A_1_R targeting remains challenging due to high structural conservation within the orthosteric binding site (7) and on-target unwanted effects associated with prototypical agonists, including bradycardia (8). Allosteric sites typically show greater divergence across subtypes, and A_1_R PAMs can display exquisite spatiotemporal specificity due to the local increase in endogenous adenosine associated with tissue injury and/or stress. We have shown that the A_1_R PAM, **1** (MIPS521), has on-target therapeutic efficacy and negligible unwanted effects in a preclinical model of neuropathic pain (9). As such, A_1_R PAMs represent a promising therapeutic avenue.

Following recent breakthroughs in GPCR structural biology, structure-based virtual screening has been successfully applied to identify orthosteric GPCR ligands (10). However, few studies have reported virtual screens for GPCR allosteric ligands (11–13). Recently, we solved a 3.2 Å cryoelectron microscopy structure of active A_1_R complexed with G_i2_ heterotrimer and cobound to adenosine and **1**, revealing an extrahelical binding pocket facing the lipid bilayer (9). Access to this structure provided a unique opportunity to harness virtual screening for A_1_R PAM discovery. However, binding sites located in the receptor interface with the membrane are challenging to model. Molecular docking algorithms typically assume the protein is surrounded by an aqueous solvent, leading to a poor description of ligand binding to this extrahelical pocket.

Herein, we develop a virtual screening strategy to discover allosteric ligands of extrahelical binding pockets, with a focus on the A_1_R. Informed by molecular dynamics (MD), an ensemble docking approach screened 160 million commercially available compounds against simulation snapshots of the lipid membrane-embedded allosteric pocket. Top-ranked compounds were assessed experimentally to evaluate PAM activity. Structure-guided optimization of virtual screening hits identified two A_1_R PAMs with activity in recombinant expression systems and primary cortical neurons. Our approach provides a validated strategy for structure-guided GPCR allosteric drug development.

## Results

### Docking Approach for Extrahelical GPCR Binding Sites.

**Fig. 1.**
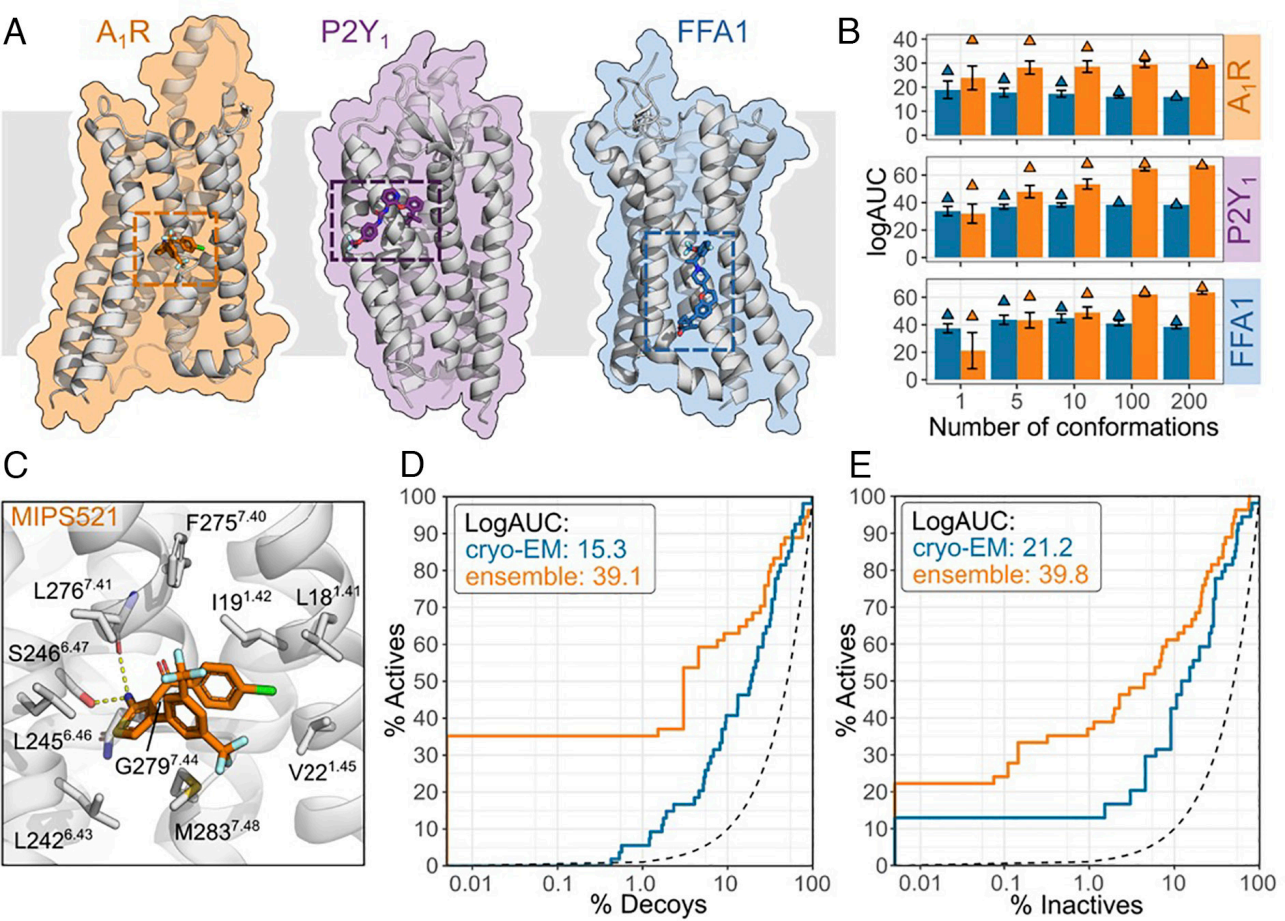
Molecular docking at GPCR extrahelical binding sites. (*A*) Allosteric binding site location of A_1_R (PDB accession code: 7LD3), P2Y_1_ (PDB accession code: 4XNV), and FFA_1_ (PDB accession code: 5TZY). The receptors are represented as gray cartoons and the ligands as sticks. The phospholipid bilayer location is shown by the gray square. (*B*) Virtual screening performance of ensemble docking using implicit and explicit membrane modeling, shown in blue and orange, respectively. In the implicit membrane model, the dielectric constant for the region surrounding the binding site was reduced from ε = 80 to ε = 2 in order to represent the hydrophobic core of the membrane. The explicit membrane model used lipid bilayer conformations (1 to 200 snapshots) obtained from MD simulations. Ligand enrichment over property-matched decoys was assessed using logAUC values. The logAUC values obtained with the top-enriching structures are shown with triangles. Error bars correspond to the SD. For FFA1, conformations obtained from two structures (PDB accession codes 5TZY and 5KW2) were combined. (*C*) Structure of A_1_R in complex with the PAM **1** (MIPS521). **1** is shown as sticks with orange carbon atoms. Side chains interacting with the PAM are shown as gray sticks (PDB accession code: 7LD3). ROC curve for the enrichment of A_1_R PAMs over property-matched decoys (*D*) and experimentally confirmed inactive compounds (*E*). Enrichment curves obtained from docking to the experimental structure or an ensemble of five MD snapshots that included the lipid bilayer are shown in blue and orange, respectively. The dashed black line represents the enrichment expected from random selection.

A large fraction of allosteric ligands occupying extrahelical pockets are exposed to the membrane, and these interactions will play an important role in binding. However, as molecular docking scoring functions have been developed for binding pockets surrounded by water, these allosteric sites may be poorly described by standard protocols. Two techniques were explored to represent the membrane environment in docking calculations for extrahelical binding sites. In the first model, the dielectric constant of the region surrounding the binding site was reduced from a value representing aqueous solution (ε = 80) to 2, which more accurately describes the hydrophobic core of the membrane. The second model treated the membrane explicitly using a lipid bilayer obtained from MD simulations. In both models, binding energy was calculated using a physics-based scoring function, which included the ligand interaction energy and a term accounting for the complete desolvation of the ligand upon binding to the extrahelical site. The two approaches were evaluated using a set of three GPCRs for which experimentally determined structures of complexes with allosteric modulators were available (Fig. 1*A*). The allosteric modulators occupied three distinct extrahelical binding pockets in the A_1_R, P2Y_1_, and FFA_1_ receptors. For each complex, we first simulated the receptor and allosteric modulator restrained to the experimental structure. An ensemble of 200 lipid conformations per structure was extracted and prepared for docking with DOCK3.7 (14). Docking performance with the two membrane representations was assessed using a database of known allosteric modulators and property-matched decoys. Ligand enrichment was then quantified using receiver operating characteristics (ROC) curves and the adjusted LogAUC metric (15). LogAUC values close to zero are expected for random selection from the database, whereas positive values indicate the docking scoring function enriches actives over decoys. The performance of the membrane representations was first assessed using a single MD snapshot, and the average LogAUC values were positive for all binding sites (Fig. 1*B*). The explicit membrane representation resulted in the best ligand enrichment for the A_1_R, but the implicit model was better for the P2Y_1_ and FFAR_1_ receptor. Next, ensembles of between 2 and 200 MD snapshots were used in the ligand enrichment calculations, which accounted for plasticity of the lipid bilayer in the explicit membrane model. Increasing the number of snapshots in the ensemble improved the ligand enrichment for the P2Y_1_ and FFA_1_ receptors. The explicit membrane model with 200 lipid conformations was the best alternative for all three binding sites, and strong enrichment of allosteric modulators was obtained. Based on these benchmarking calculations, the prospective virtual screen against the A_1_R was performed using explicit membrane representation.

### Docking Screens of Chemical Libraries for A_1_R Allosteric Modulators.

Molecular docking screens of commercially available chemical libraries were performed against the cryo-EM structure of the A_1_R in a complex with adenosine and **1** (MIPS521; PDB ID: 7LD3; Fig. 1*C*) (9). **1** (MIPS521) binds to an extrahelical site and interacts with the protein through hydrogen bonds with the side chain of S246^6.47^ and the backbone of L276^7.41^ and van der Waals interactions with the pocket formed by residues located on TM1, TM6, and TM7 (superscript refers to Ballesteros–Weinstein nomenclature) (16). Notably, the orthosteric and allosteric sites exhibit distinct characteristics that could influence the likelihood of identifying ligands through structure-based virtual screening. The orthosteric site is deep and enclosed, whereas the allosteric pocket is shallow and open (*SI Appendix,* Fig. S1). Molecular docking was performed using an ensemble of lipid conformations obtained from MD simulations of the complex embedded in a lipid bilayer. A set of 250 snapshots was extracted from a 20 ns simulation, and enrichment of PAMs was evaluated using property-matched decoys (17) and experimentally verified inactive compounds. The five MD snapshots with the best PAM enrichment using property-matched decoys were then used in the prospective virtual screen. The shape of the allosteric site in these snapshots was similar to that of the cryo-EM structure, with slight changes in side chain conformations (*SI Appendix,* Fig. S2). This ensemble of structures resulted in excellent enrichment of allosteric ligands (LogAUC = 39 and 40 for property-matched decoy and inactive compounds, respectively) and outperformed docking to the cryo-EM structure (LogAUC = 15 and 21, respectively) (Fig. 1 *D* and *E*).

Commercially available compounds from the ZINC database (18) were used to design a screening library. Comparison of the PAM physicochemical properties and experimentally confirmed inactive compounds revealed that actives were generally more lipophilic, likely due to the characteristics of the binding site. For this reason, molecules with LogP values between 2.5 and 4.5 from the fragment (MW < 250 Da) and lead-like (250 < MW <350 Da) libraries from the ZINC15 database (4.4 and 156 million compounds, respectively) were included. Several thousand orientations were sampled in the binding site for each of the 160 million molecules with the protein kept rigid, resulting in the evaluation of trillions of predicted complexes using the DOCK3.7 scoring function. The top 0.5% ranked compounds of the fragment and lead-like libraries were first filtered by excluding molecules that did not form the hydrogen bonds with S246^6.47^ and L276^7.41^ observed in the complex with **1** (MIPS521). Molecules were subsequently clustered by 2D structure similarity, and only the best-scoring representative from each cluster was retained to increase compound diversity. The top 1,000 cluster representatives of each library were visually inspected. A set of 26 compounds, comprising 16 fragment- and 10 lead-like compounds, were finally selected for experimental evaluation based on interactions with the allosteric binding site (*SI Appendix,* Table S1).

### Evaluation of the Effect of Predicted Compounds on A_1_R Pharmacology.

**Fig. 2.**
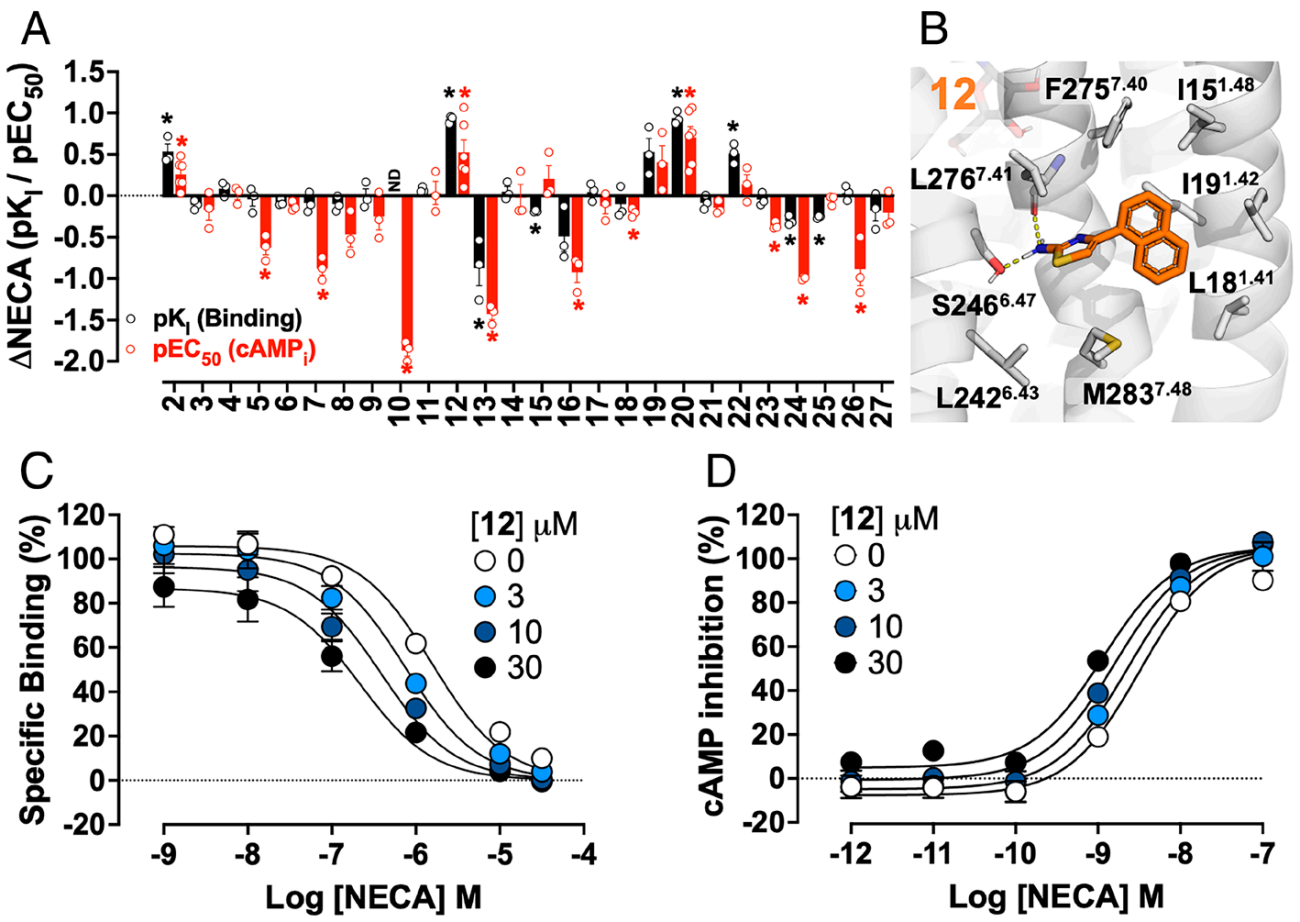
Influence of virtual screening hits on NECA A_1_R pharmacology. (*A*) The change in NECA affinity (ΔpK_I_; black) and potency (ΔpEC_50_; red) in the presence of 30 μM virtual screening hit compounds in A_1_R-FlpInCHO cells was assessed using [^3^H]DPCPX competition binding or cAMP inhibition, respectively. (*B*) Predicted binding mode for virtual screening compound **12** (orange carbon sticks). A_1_R is shown as a gray cartoon with key residues in sticks. Hydrogen bonds are indicated with yellow dashed lines. Interaction profile of NECA and **12** in [^3^H]DPCPX competition binding (*C*) and inhibition of forskolin-stimulated cAMP accumulation (*D*) in the absence and presence of **12** (3, 10, and 30 μM) in A_1_R-FlpInCHO cells. Data represent the mean ± SEM from n = 3 to 4 individual replicates performed in duplicate. *P < 0.05, one-sample *t* test compared to a hypothetical value of 0. ND denotes not determined due to near complete inhibition of [^3^H]DPCPX binding.

A_1_R PAMs that enhance adenosine binding affinity and functional potency with minimal allosteric agonism can selectively potentiate adenosine-mediated A_1_R stimulation, thereby limiting chronic receptor activation, which may be therapeutically unfavorable. The initial experimental evaluation aimed to establish whether the 26 in silico hit compounds could modulate the A_1_R binding affinity and functional potency of 5′-*N*-ethylcarboxamidoadenosine (NECA), a nonselective adenosine receptor orthosteric agonist. NECA affinity and potency were quantified using whole-cell [^3^H]DPCPX competition binding and inhibition of forskolin-stimulated cAMP accumulation assays in human A_1_R-FlpINCHO cells, respectively (*SI Appendix,* Fig. S3 and Table S1). NECA affinity (pK_I_) was significantly enhanced by 30 μM of **2**, **12**, **20**, and **22** (Fig. 2*A*; *P* < 0.05; one-sample *t* test compared to zero). NECA potency (pEC_50_) was significantly enhanced in the presence of 30 μM **2**, **12,** and **20** (Fig. 2*A*; *P* < 0.05; one-sample *t* test compared to zero). **12** and **20** were identified as A_1_R PAMs that mediated the most significant increase in orthosteric agonist affinity and potency.

We next evaluated the potential for allosteric agonism. In the absence of NECA, **1** (MIPS521; 30 μM) stimulated a significant functional response (*P* < 0.05; one-sample *t* test), approximately 80% of the NECA maximal response (83% vs. 103% inhibition of 3 μM forskolin-stimulated cAMP accumulation, respectively), whereas 30 μM **12** and **20** had no significant effect (*P* < 0.05; one-sample *t* test; *SI Appendix,* Fig. S4*A*). A modest but significant decrease in [^3^H]DPCPX binding (1 nM) was observed in the presence of 30 μM **12** and **20** (*P* < 0.05; one-sample *t* test; *SI Appendix,* Fig. S4*B*). Negative cooperativity with orthosteric antagonists is consistent with other A_1_R PAMs (19), suggesting **12** and **20** prefer active A_1_R states. While **20** has a very similar structure to known A_1_R PAMs, ChEMBL127146 being the closest with a Tanimoto coefficient (Tc) score of 0.76, **12** has a nonaminothiophene scaffold and a Tc score of 0.4 for the most similar A_1_R compound in the CHEMBL database (*SI Appendix,* Table S1). As such, **12** (Fig. 2*B*) was a promising hit compound for further characterization as an A_1_R PAM.

Compound **12** modulation of NECA potency and affinity was further investigated. Whole-cell [^3^H]DPCPX interaction binding assays were performed using increasing concentrations of the orthosteric agonist, NECA, and the allosteric ligands **12** (Fig. 2*C*) and **1** (*SI Appendix,* Fig. S5*A*), a known A_1_R PAM with high positive cooperativity (9). Data fit an allosteric ternary complex model, allowing comparison of allosteric ligand affinity (pK_B_) and binding cooperativity with the orthosteric agonist (logα_I_) for **12** and the reference PAM, **1** (MIPS521). Micromolar affinity for the A_1_R allosteric site was observed for **1** and **12**, approximately 8 and 25 μM, respectively (pK_B_: **1** 5.18 ± 0.21; **12** 4.63 ± 0.32; n = 4). Positive cooperativity between the PAM and orthosteric agonist NECA was approximately 100-fold for **1** and 10-fold for **12** (logα: **1** 2.09 ± 0.22; **12** 0.99 ± 0.25; n = 4). **12** and **1** modulation of NECA-mediated inhibition of forskolin-stimulated cAMP accumulation in A_1_R-FlpInCHO cells fit an operational model of allosterism, enabling estimation of allosteric ligand efficacy (logτ_B_) and functional cooperativity (logαβ) between the allosteric ligand and NECA (Fig. 2*D* and *SI Appendix,* Fig. S5*B*). At the A_1_R, **12** enhanced NECA potency approximately fivefold (logαβ: 0.68 ± 0.10; n = 4), similar to the positive cooperativity observed for **1** (logαβ: 0.78 ± 0.15; n = 4). In the absence of NECA, **1** displayed high allosteric agonism, mediating a concentration-dependent increase in the inhibition of 3 μM forskolin-stimulated cAMP accumulation (logτ_B_: 0.56 ± 0.06; n = 4; *SI Appendix,* Fig. S5*B*), whereas **12** displayed minimal allosteric agonism (logτ_B_: −0.65 ± 0.12; n = 4; Fig. 2*D*). As such, while **12** and **1** enhanced agonist affinity and potency at the A_1_R, **12** had lower allosteric agonism (logτ_B_) than **1**, which may be therapeutically beneficial to prevent unwanted A_1_R activation in the absence of endogenous adenosine.

### Structure-Guided Optimization of A_1_R PAM.

**Fig. 3.**
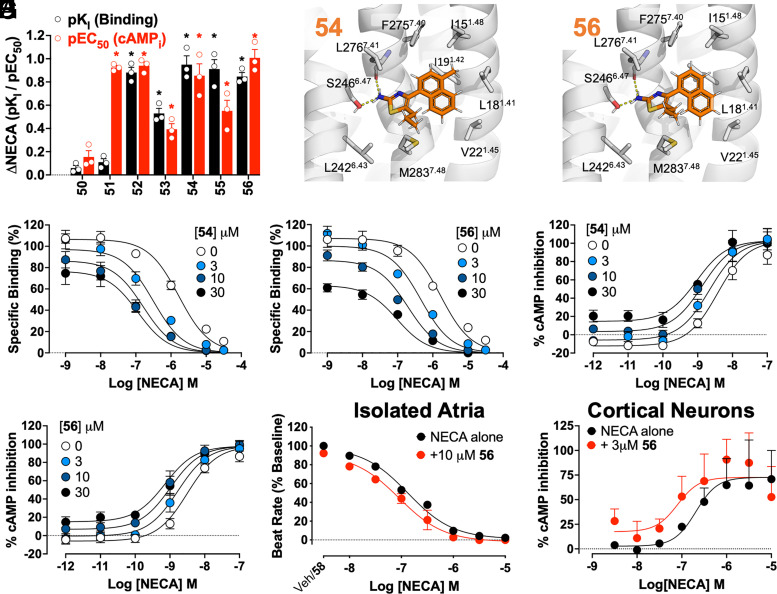
Structure-guided optimization of A_1_R PAMs. (*A*) The change in NECA affinity (ΔpK_I_; black) and potency (pEC_50_; red) in the presence of 30 μM **50–56** in A_1_R-FlpInCHO cells, assessed in [^3^H]DPCPX competition binding or inhibition of forskolin-stimulated cAMP accumulation, respectively. Data represent the mean ± SEM from n = 3 to 4 individual replicates performed in duplicate. ^∗^*P* < 0.05, one-sample *t* test compared to a hypothetical value of 0. The predicted binding mode of A_1_R PAMs **54** (*B*) and **56** (*C*). Ligands are shown as sticks with carbon atoms in orange, A_1_R as a gray cartoon with key residues as sticks, and hydrogen bonds in yellow dashed lines. [^3^H]DPCPX interaction binding with NECA and **54** (*D*) and **56** (*E*), and NECA inhibition of forskolin-stimulated cAMP accumulation with **54** (*F*) and **56** (*G*) in A_1_R-FlpInCHO cells. Data represent the mean ± SEM from n = 3 to 4 individual replicates performed in duplicate. (*H*) The influence of **56** on the NECA-mediated decrease in the contractile rate of isolated rat atria. (*I*) The influence of **56** on NECA-mediated inhibition of forskolin-stimulated cAMP accumulation in primary mouse cortical neurons. Data represent the mean ± SEM from n = 3 to 4 individual replicates performed in duplicate.

Interaction studies characterized **12** as an A_1_R PAM with low intrinsic efficacy and modest affinity and cooperativity, and this compound was used as a starting point for hit-to-lead optimization. Substructure searches for compounds containing a 2-aminothiazole moiety in the ZINC database identified 6,729 analogs. Similarity searches (Tc ≥ 0.5, ECFP4 molecular fingerprints) identified an additional 333 analogs. The two sets of analogs were docked to the allosteric pocket, and the predicted binding modes were visually inspected. A set of 22 compounds (**28–49**) with a thiazole core were selected for experimental evaluation (Table 1). Of these compounds, 30 μM **34**, **35**, **37**, **38**, and **44** increased NECA potency and affinity between approximately 3- to 10-fold in inhibition of cAMP accumulation and [^3^H]DPCPX binding assays (Table 1 and *SI Appendix,* Fig. S6), supporting an A_1_R PAM mechanism of action. Two compounds with the greatest effect, **37** and **38** differ only by the position of a methyl group localized at either the C4 position of the naphthalene moiety (20) or at the C5 position of the thiazole (21). We further explored the role of these two positions in the allosteric effect by combining different substituents, resulting in the design and in-house synthesis of seven compounds. Briefly, **50–56** were synthesized starting from naphthalene derivatives. Friedel-Craft aromatic acylation with acyl chlorides catalyzed by AlCl_3_ afforded ketone intermediates. Then, selective *alpha-*bromination using pyridinium hydrotribromide afforded alpha bromo ketone intermediates, which were condensed with thiourea to afford the target products **50–56** (Table 1 and *SI Appendix*, *SI Materials and Methods*). Of the seven compounds, six (**51–56**) significantly enhanced NECA potency, and five (**52–56**) significantly increased NECA affinity (Fig. 3*A* and Table 1 and *SI Appendix,* Fig. S8). Generally, the rigid alkyl group with 5-propyl was preferable for enhancing NECA affinity and potency (**53–56**). Relative to the virtual screening hit **12**, modest improvements were observed for **54** and **56**. **54** (Fig. 3*B*) had an approximate threefold improved affinity (pK_B_: 5.15 ± 0.12; n = 4), while both **54** and **56** (Fig. 3*C*) had greater binding cooperativity (logα: **54** 1.15 ± 0.10; **56** 1.30 ± 0.18) and greater functional cooperativity (logαβ: **54** 0.84 ± 015; **56** 0.92 ± 0.12) (Fig. 3 *D*–*G*). **54** and **56** exhibited minimal allosteric agonism, as evidenced by low efficacy (τ_B_) values (logτ_B_: **54** −0.37 ± 0.10; **56** −0.07 ± 0.06), which may provide a potential therapeutic advantage of preventing chronic receptor activation in the absence of endogenous agonist.

**Table 1.** Structure-guided optimization of hit A_1_R PAM from virtual screening

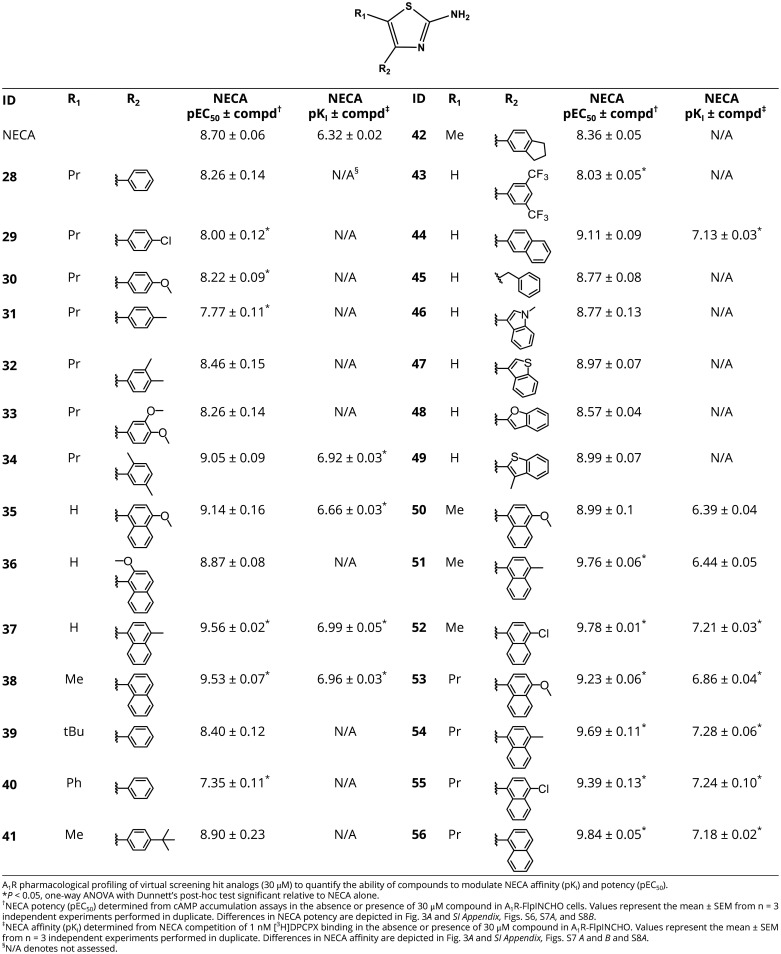

### The Influence of Binding Site Mutations on A_1_R PAMs.

We explored the predicted binding pocket for **54** (Fig. 3*B*) and **56** (Fig. 3*C*) using site-directed mutagenesis of S246^6.47^A and G279^7.44^A, two residues that interact with **1** (9). Whole-cell [^3^H]DPCPX interaction binding was performed in the absence or presence of **54** or **56**, with increasing concentrations of NECA, in wild-type and mutant A_1_R-FlpINCHO cells (*SI Appendix,* Fig. S9). In contrast to the effect on **1** (9), S246^6.47^A did not significantly decrease the affinity or cooperativity of **54** and **56** (*SI Appendix,* Table S2), suggesting interactions with the side chain of S246^6.47^ are not essential for the binding of these two PAMs. G279^7.44^A significantly decreased allosteric cooperativity with the orthosteric agonist but did not influence the affinity of **54** and **56** (*SI Appendix,* Table S2). The G279^7.44^A effect on cooperativity alone highlights that while **54** and **56** do not appear to have the same interaction profile as **1**, these compounds remain sensitive to mutations at the extrahelical region.

### Selectivity of PAM Activity.

Compounds **54** and **56** agonism and allosteric modulation in A_1_R-FlpINCHO cells were compared with effects in nontransfected FlpINCHO cells, and FlpINCHO cells expressing the A_2A_R, A_2B_R, A_3_R, or M_2_ receptor (M_2_R). While in A_1_R-FlpINCHO cells, **54** and **56** alone stimulated a modest functional response, no appreciable activity was observed in the presence of **54** or **56** (30 μM) in FlpINCHO cells expressing the A_2A_R, A_2B_R, and A_3_R (*SI Appendix,* Fig. S10). In contrast to the PAM activity observed at the A_1_R, **54** and **56** (10 µM) had no significant effect on NECA potency at the A_2A_R, A_2B_R, or A_3_R (*SI Appendix,* Fig. S10 *B–D*; one-way ANOVA, Dunnett post hoc test). **54** and **56** had no significant effect in FlpINCHO cells expressing the M_2_ receptor (*SI Appendix,* Fig. S10*E*) (22), another Family A GPCR. Similarly, **54** and **56** (10 µM) had no significant effect in nontransfected FlpINCHO cells (*SI Appendix,* Fig. S10*F*). As such, the PAM activity of **54** and **56** appears to be selective for the A_1_R subtype.

### A_1_R Allosteric Modulation of Agonist Responses in Rodent Primary Cortical Neuron and Atria.

A_1_R PAM activity for **56** was assessed in more physiologically relevant cells, specifically rodent atria and cortical neurons (Fig. 3 *H* and *I*). NECA stimulated A_1_R-mediated inhibition in rat atrial beat rate with a potency (EC_50_) of approximately 0.13 μM. **56** (10 μM) had no significant effect on NECA potency for decreasing rat atrial beat rate (*P* > 0.05; unpaired *t* test) (Fig. 3*H*). In cortical neurons, NECA stimulated A_1_R-mediated inhibition of cAMP accumulation with a potency (EC_50_) of approximately 0.2 μM (Fig. 3*I*). Consistent with recombinant cell data, **56** (3 μM) significantly enhanced NECA potency for this canonical signaling pathway in primary neuron cultures (*P* < 0.05; unpaired *t* test). Bradycardia is one of the major side effects of prototypical A_1_R agonists. Therefore, **56** represents a useful pharmacological tool to probe the therapeutic potential of A_1_R PAMs in the brain with minimal unwanted cardiac effects.

## Discussion

Allosteric modulators of GPCRs have considerable therapeutic potential. One major limitation of rational drug design has been the lack of structural information regarding the binding sites of many allosteric modulators. The recent determination of a cryo-EM structure of the A_1_R complexed with an allosteric modulator enabled molecular docking screens for structurally distinct PAMs. In this study, virtual screening protocols for docking to extrahelical binding sites of GPCRs that consider the lipid membrane environment were first developed and proved amenable to virtual screening. Several PAMs were discovered, and structure-guided optimization identified **54** and **56** as promising A_1_R allosteric modulators. These PAMs displayed moderate affinity and cooperativity, low allosteric agonism, and subtype-selective PAM activity. The PAM activity of **56** was maintained in primary cortical neurons, enabling future studies to evaluate A_1_R PAMs as a potential therapeutic approach to treat neurological conditions such as epilepsy (9, 23).

Recent structural studies revealed allosteric binding pockets in diverse regions of class A GPCRs, including extracellular, intracellular, and transmembrane sites. Unexpectedly, compound **1** (MIPS521) and allosteric modulators of several other GPCRs were found to interact with extrahelical binding sites (24). These shallow and lipid-facing pockets are challenging to model computationally and require the development of virtual screening techniques to account for the membrane. In this study, a more accurate description of ligand binding to extrahelical pockets was achieved by modifying the molecular docking scoring function and modeling the lipid membrane based on MD simulation snapshots. Our approach markedly improved virtual screening performance for allosteric pockets identified in three different class A GPCRs (10). The same approach can now be applied to other GPCRs, or other transmembrane-spanning proteins, for which allosteric modulators bound to extrahelical pockets are predicted (25) or structurally determined (24).

A_1_R is an attractive therapeutic target, and drug discovery efforts have identified thousands of ligands representing diverse chemical scaffolds. However, the majority are orthosteric ligands, and only a small set of allosteric modulators have been discovered. Development of PAMs has primarily focused on the 2-amino-3-benzoylthiophene scaffold identified over 30 y ago (26), but the physicochemical properties of these compounds remain suboptimal for drug development (27). The cryo-EM A_1_R structure in complex with **1** (MIPS521) revealed the binding site of this compound class (9). From 26 top-ranked compounds selected from our allosteric binding docking, three compounds enhanced A_1_R NECA affinity and potency, corresponding to an 11.5% hit rate. However, a corrected 3.8% hit rate corresponds to the single structurally distinct compound **12** characterized as an A_1_R PAM. Recently performed virtual screens targeting GPCR orthosteric binding sites achieved hit rates as high as 82% and identified nanomolar antagonists (10). These observations clearly illustrate shallow extrahelical pockets are considerably more difficult targets than the orthosteric site, which is deeply buried in the receptor and evolved to bind small molecules with high affinity. Encouragingly, one of the discovered PAM, compound **12**, represented a 2-aminothiazole scaffold, supporting the ability of structure-based screening approaches to identify structurally distinct starting points for the development of allosteric drugs. Previously, certain 2-aminothiazoles were reported as A_1_R allosteric enhancers (28). However, this classification was not supported by a subsequent study (29). The current study validated **12** as an A_1_R PAM with micromolar affinity for the allosteric site. While the positive cooperativity observed between **12** and NECA was lower than for **1** (MIPS521), **12** displayed relatively low agonism compared to known A_1_R PAMs. Excessive receptor activation through high allosteric agonism may cause unwanted effects, and therefore **12** was an excellent candidate for further optimization.

Structure-guided design of 29 analogs based on the most promising hits from the virtual screen led to the discovery of a series of 2-aminothiazole-based PAMs. Medicinal chemistry efforts starting from **12** retained cooperativity with modest improvement in affinity. Allosteric ligand SAR exploration is notoriously challenging, which may in part, be attributable to targeting shallow nonconserved pockets. The 2-aminothiazole-based A_1_R PAMs were predicted to bind in the same pocket as **1** (MIPS521), and the interactions in the site were explored using a single alanine substitution of two residues. While the G279^7.44^A mutation displayed a significant reduction in the binding cooperativity of **54** and **56** with the orthosteric agonist, S246^6.47^A had no significant effect. In contrast, these binding site mutations caused a small (four- to seven-fold) but significant reduction in **1** affinity (9). Hence, although mutations in this region influenced the 2-aminothiazole-based PAMs, further mutational analysis or structural studies would be required to determine the binding mode of these compounds within the allosteric pocket.

One of the major advantages of targeting allosteric sites is that the sequence variability in these pockets facilitates the development of subtype-selective compounds. In agreement with this idea, **54** or **56** did not activate the other three adenosine receptor subtypes or the M_2_ receptor, a distinct family A GPCR. Also, the compounds did not affect agonist potency at the other adenosine receptor subtypes (A_2A_R, A_2B_R, A_3_R) or the M_2_ receptor. The 2-aminothiazole substructure was previously shown to have increased frequency as a fragment-based drug discovery screening hit at a concentration of 200 μM (30). However, consistent with hit analysis from high-throughput screens, which used 2-aminothiazole concentrations between 10 and 25 μM, our 2-aminothiazole-based A_1_R PAMs were not frequent hitters, only displaying PAM activity at the A_1_R.

A comparison of the PAM physicochemical properties revealed that compounds **54** and **56** have cLogP values of 4.49 and 4.14, respectively, an improvement over **1** (MIPS521), which has a cLogP of 5.88 (calculated using Percepta^®^ software). Compounds **54** and **56** are compliant with Lipinski’s rules and have predicted blood–brain barrier permeability (31). Moreover, the thiophene core present in **1** (MIPS521) is generally considered a “structural alert” due to the propensity to undergo conversion to reactive metabolites (32). Consistent with this, **1** (MIPS521) was previously reported to undergo rapid metabolism during in vitro liver microsome assays (9). Conversely, the thiazole core in compounds **54** and **56** is more robust and present in several clinical agents. A thiazole was present in eight small molecule drugs approved by the FDA in the decade from January 2013 to December 2023 (33), and the thiazole was the 14th most common ring system present in FDA-approved small molecule drugs in analysis performed up to January 2020 (34). As such, **54** and **56** represent promising candidates for further optimization.

The best compounds, **54** and **56**, had low micromolar affinity and improved binding cooperativity. Despite the modest affinity, the positive cooperativity of our 2-aminothiazole-based A_1_R PAMs will increase apparent affinity ~10-fold in the presence of endogenous adenosine, which is typically elevated under disease conditions (6, 27). Moreover, a similar affinity of **1** (MIPS521) was sufficient to show in vivo analgesic efficacy in a rat model (9). To further assess the therapeutic potential of the discovered PAMs, we characterized **56** pharmacology in primary cortical neurons. In primary cortical neurons, **56** significantly enhanced orthosteric agonist potency at the canonical A_1_R signaling pathway. Compared to the known A_1_R PAM **1** (MIPS521), the cooperativity of **54** and **56** was lower. However, a study on M_1_ receptor PAMs suggested low to moderate cooperativity between M_1_ PAMs and acetylcholine may reduce adverse effects (35). At the A_1_R, bradycardia often represents an unwanted A_1_R-mediated effect. Such on-target unwanted effects have hindered the transition of adenosine receptor therapeutics into the clinic (27). Encouragingly, **56** did not significantly enhance NECA potency for reducing atrial beat rate. The lack of effect of **56** on the atrial beat rate indicates it could be a useful pharmacological tool to probe the therapeutic potential of enhancing endogenous adenosine activity at the A_1_R in the brain without causing unwanted effects on the heart.

Allosteric modulation of GPCRs is a promising approach to overcome many limitations of orthosteric drugs. In this study, we demonstrate that access to structural information regarding the binding site of an A_1_R PAM enabled allosteric drug design, which led to the discovery of structurally distinct modulators with a promising pharmacological profile. The rapidly increasing number of experimental structures of GPCRs combined with computational methods to identify novel binding sites could further expand the number of possible targets. Our general strategy to identify ligands of extrahelical binding sites by structure-based virtual screening of large chemical libraries can be applied to numerous therapeutic targets in the GPCR family, and to transmembrane proteins more broadly, to accelerate allosteric drug development.

## Materials and Methods

### Materials.

Chinese hamster ovary (CHO) Flp-IN™ (FlpInCHO) cells, Dulbecco’s modified Eagle’s medium (DMEM), and hygromycin B (Hygrogold™) were purchased from Invitrogen (Carlsbad, CA). Fetal bovine serum (FBS) was purchased from ThermoTrace (Melbourne, VIC, Australia). Adenosine deaminase (ADA) was purchased from Roche (Basel, Switzerland). AlphaScreen™ reagents, OptiPhase Supermix™ scintillation cocktail and [^3^H]DPCPX (8-cyclopentyl-1,3-dipropylxanthine, [dipropyl-2,3-^3^H(N)]; specific activity, 120 Ci/mmol) were purchased from PerkinElmer (Boston, MA). SLV320 ((trans-4-[(2-phenyl-7*H*-pyrrolo[2,3-d]pyrimidin-4-yl)amino]cyclohexanol)) was purchased from Tocris Bioscience (Bristol, UK). Compounds were purchased from commercial vendors or synthesized in-house (*SI Appendix*, Table S3 and *SI Materials and Methods*). All other reagents were purchased from Sigma-Aldrich and Tocris and were analytical grade. **1** (MIPS521) was synthesized as described previously (36).

### MD Simulations.

MD simulations were performed using the experimentally determined structures of the A_1_R in complex with **1** (MIPS521) (PDB accession code: 7LD3), P2Y_1_ receptor in complex with BTPU (PDB accession code: 4XNV), and FFA_1_ receptor in complex with AP8/MK-8666 and 6XQ (PDB accession codes: 5TZY and 5KW2, respectively). The A_1_R structure was determined in complex with a G protein, which was removed to reduce the computational cost of the MD simulations. Each complex was embedded in a POPC membrane bilayer using the CHARMM-GUI server and then solvated in a 0.15 M concentration of neutralizing sodium and chloride ions (37, 38). Ionizable residues were protonated according to the most probable state in an aqueous solution at pH 7. MD simulations of the A_1_R were performed in NAMD whereas simulations of the P2Y_1_ and FFA_1_ receptors were performed in GROMACS (20, 21). The CHARMM36m force field (39) was used in all cases and ligand parameters were derived using the *CHARMM* General Force Field (*CGenFF*) (40). Each system was equilibrated by gradually increasing the temperature to 303.15 K and releasing restraints on membrane bilayer atoms. During the production phase, the heavy atoms of the protein and ligand were restrained to initial coordinates using a harmonic potential with a force constant of 5 kcal/Å^2^. MD trajectories of 20 ns for the A_1_R and 100 ns simulations for the other two receptors were then generated. A longer simulation time was used for the P2Y_1_ and FFA_1_ receptors because the bound allosteric modulators and pockets were larger, which may require more sampling of the lipid bilayer. For each system, 200 snapshots were extracted from the simulations and used in the molecular docking calculations.

### Molecular Docking Screens.

Molecular docking was performed with the program DOCK3.7 (14) using an ensemble of structures from the receptor MD simulations. The atomic coordinates of the receptors, the cocrystalized ligands, and POPC molecules within 14 Å of the allosteric ligand were extracted from the MD snapshots and aligned to the experimentally determined structure. Protonation states of ionizable residues (Asp, Glu, Lys, Arg, and His) and coordinates of polar hydrogen were kept from the MD simulations. The dipole moments of the S246^6.47^ side chain and carbonyl of the L276^7.41^ backbone in the A_1_R, L102^2.55^ backbone carbonyl in the P2Y_1_ receptor, and the side chains of Y44^2.42^, Y114^ICL2^, and S123^4.42^ in the FFA_1_ receptor were increased to favor hydrogen bonding interactions with these residues, as described previously (41). The DOCK3.7 flexible ligand docking algorithm uses matching spheres to define the binding site, and 45 matching spheres were generated based on the coordinates of the allosteric ligand. The scoring grids needed to calculate the binding energy (electrostatic, van der Waals and ligand desolvation terms) were prepared using DOCK3.7. The electrostatics and van der Waals grids were prepared using a united-atom AMBER force field (42). Three different grid generation protocols were used: default parameters, implicit membrane, and explicit membrane. The three types of grids differed in the treatment of the membrane bilayer, the electrostatic grid parameters, and the ligand desolvation model. In the docking calculations with default settings, the lipids were removed, and the receptor was surrounded by a continuum with a dielectric constant of 80. In this case, the default scoring function was also applied to calculate the ligand desolvation energy. In the implicit membrane protocol, the dielectric constant of the solvent was reduced to a value of 2, and ligands were treated as fully desolvated in the complex by the docking scoring function. Finally, in the explicit membrane protocol the heavy atoms and polar hydrogens of the POPC molecules were kept from the MD snapshots, and ligands were treated as fully desolvated upon binding. Molecular docking was carried out to the experimentally determined structure and 200 MD snapshots, and the receptor structure and lipid bilayer were treated as rigid in the calculations. For each docked molecule, the lowest energy docking pose was refined by using 100 steps of rigid-body minimization. Docking performance was assessed based on the enrichment of known allosteric modulators in a database of ligands and decoys. Ligands and decoys were prepared for docking using the ZINC database protocol (18). A set of 55 allosteric modulators obtained from previous experimental screens (43–47) was used for the A_1_R whereas 137 and 63 allosteric modulators from the ChEMBL database (Molecular weight < 500 Da) were used for the P2Y_1_ and FFA_1_ receptors, respectively. For each ligand, 40 to 50 property-matched decoys were generated using the DUD-E protocol (17). For A_1_R, enrichment of ligands was also evaluated using a set of experimentally inactive compounds (66 molecules), which were obtained from previous studies (26, 44, 46). Ligand enrichment was analyzed using ROC curves and quantified using the adjusted logAUC metric (15).

In the prospective virtual screen against the A_1_R allosteric site, two commercial chemical libraries from the ZINC15 database (18) (a fragment and lead-like library of 4.4 and 156 million compounds, respectively) were screened against five MD snapshots using explicit membrane representation. The five structures were selected based on the ligand enrichment by 200 MD snapshots using property-matched decoys. From the top 0.5% ranked compounds of each screened library, molecules that did not interact with the S246^6.47^ side chain and backbone carbonyl of L276^7.41^ were first excluded. For each compound, the best energy pose was then selected among the five MD snapshots. To increase diversity among the compounds considered for experimental evaluation, the top-ranked molecules were clustered by 2D similarity (ECFP4 fingerprints calculated using Rdkit (https://www.rdkit.org), Tanimoto similarity threshold of 0.5). Only the best-scoring representative of each cluster was retained, and compounds were selected from the top 1,000 clusters.

### Cell Culture.

FlpInCHO cells, nontransfected (NT) or stably transfected with human wild-type (WT) A_1_R, A_2A_R, A_2B_R, A_3_R, and M_2_R or mutant A_1_R containing a triple human influenza hemagglutinin (HA) N-terminal tag (3xHA-A_1_Rs) were generated as previously described (9, 22, 48–51). Cells were maintained in DMEM supplemented with 10% FBS and 500 μg/mL hygromycin-B and were grown at 37 °C in a humidified incubator containing 5% CO_2_. Isolation and maintenance of mixed-sex embryonic cortical neurons from Swiss mice were performed as described previously. Use of animals was approved by the Monash Institute of Pharmaceutical Sciences Animal Ethics Committee under project number 13003 (52). Growth medium was replaced with serum-free DMEM-high glucose at least 6 h prior to assaying.

### cAMP Accumulation Assay.

FlpInCHO cell lines and cortical neuron cells were seeded into transparent 96-well plates at 20,000 cells/well and incubated for 16 to 20 h in a humidified incubator at 37 °C in 5% CO_2_. On the day of assay, medium was removed from wells and replaced with cAMP stimulation buffer (140 mM NaCl, 5 mM KCl, 0.8 μM MgSO_4_, 0.2 mM Na_2_HPO_4_, 0.44 mM KH_2_PO_4_, 1.3 mM CaCl_2_, 5.6 mM D-glucose, 5 mM HEPES, 0.1% bovine serum albumin (BSA), 1 U/mL ADA, and 10 μM rolipram, pH 7.45). Compounds were added at either a single concentration (10 or 30 μM) or a concentration range for full interaction studies (3 to 30 μM), and plates were incubated for 30 to 60 min at 37 °C in a humidified chamber. Cells were then exposed to NECA or acetylcholine chloride at increasing concentrations (0.01 nM to 100 μM) and 3 μM forskolin (for G_i_-coupled receptors only: A_1_R, A_3_R, and M_2_R). After 30 min incubation at 37 °C, the reaction was terminated by rapid removal of buffer and the addition of 50 μL/well cold ethanol. Following ethanol evaporation, the precipitate was resuspended in 50 μL/well lysis buffer (0.1% BSA, 0.3% Tween-20, 5 mM HEPES, in MQ water; pH 7.45), and detection of cAMP was performed using LANCE^®^ or LANCE^®^ Ultra cAMP Assay kits (PerkinElmer; Boston, MA) and fluorescence measured using an EnVision^®^ plate reader (PerkinElmer; Boston, MA). Agonist concentration–response curves were normalized to the response mediated by 3 µM forskolin (0%) or buffer alone (100%).

### Whole-Cell Radioligand Binding.

[^3^H]DPCPX (8-cyclopentyl-1,3-dipropylxanthine, [dipropyl-2,3-^3^H(N)]) whole-cell binding assays were performed on FlpINCHO cells stably expressing either WT (nontagged or 3xHA-tagged) or mutant 3xHA-A_1_R. Cells were seeded into a transparent 96-well plate at 40,000 cells/well in DMEM containing 10% FBS. Following 8 h incubation in a humidified environment at 37 °C in 5% CO_2_, cells were washed and maintained in serum-free DMEM for approximately 18 h at 37 °C in 5% CO_2_. [^3^H]DPCPX competition binding on intact cells was performed at 4 °C for 12 h in a final volume of 100 μL HEPES buffer (145 mM NaCl, 10 mM D-Glucose, 5 mM KCl, 1 mM MgSO_4_, 10 mM HEPES, 1.3 mM CaCl_2_, and 15 mM NaHCO_3_, pH 7.45) containing approximately 1 nM [^3^H]DPCPX in the presence of increasing concentrations of NECA (0.3 nM to 10 μM) and/or test compounds. Nonspecific binding was defined in the presence of a saturating concentration (1 μM) of the high-affinity A_1_R antagonist, SLV320. Assays were terminated by washing twice with 100 μL/well cold phosphate-buffered saline, followed by the addition of 100 μL OptiPhase Supermix™ scintillation cocktail and bound radioactivity measured using a MicroBeta^2^ ™ plate counter (PerkinElmer).

### Isolated Rat Atrial Experiments.

Isolated rat atrial experiments were performed as previously described (53). Briefly, 10 to 16 wk old male Sprague Dawley rats were anesthetized with xylazine/ketamine and the chest cavity of the rat was opened to fully expose the heart, which was rapidly removed and placed in Krebs–Henseleit solution. The heart was cut transversely below the right and left atria to remove the ventricles, and the remaining ventricles were trimmed carefully. Atria was then mounted in an organ bath at 37 °C, bubbled with 5% CO_2_/95% O_2_, and allowed to contract spontaneously. The rate of atrial contraction was measured using a force transducer connected to a PowerLab data acquisition system. Concentration–response curves to the NECA in the presence of **56** (10 μM) or vehicle (0.1%DMSO) were constructed, as previously described (53). All experiments involving animals were approved by the Monash Institute of Pharmaceutical Sciences Animal Ethics Committee under project number 20213.

### Data Analysis.

Data were analyzed using GraphPad Prism 8.4.3 (GraphPad Software, San Diego, CA). Statistical analysis was performed using a one-sample *t* test or a one-way ANOVA, as appropriate, with significance defined as *P* < 0.05.

[^3^H]DPCPX competition binding curves were fitted to a one-site inhibitory mass action equation:

[1] $$Y=\mathrm{Bottom}+\frac{\mathrm{Top}-\mathrm{Bottom}}{1+10^{\left(X-\mathrm{Log}{\mathrm{IC}}_{50}\right)}},$$

where *Y* is the specific binding and IC_50_ is the concentration of ligand that displaces 50% of the radioligand. The top and bottom represent the maximal and minimal binding in the presence of the competitive ligand, respectively. Equilibrium dissociation constant (K_I_) values were subsequently derived from inhibition binding experiments using the Cheng–Prusoff equation:

[2] $$K_{I}=\frac{{\mathrm{IC}}_{50}}{1+\frac{\left[A\right]}{K_{D}}}.$$

Equilibrium binding interaction experiments were fitted to the following allosteric ternary complex model.

[3] $$Y=\frac{B_{\text{max}}\left[A\right]}{\left[A\right]+\left(\frac{K_{A}K_{B}}{\alpha_{A}\left[B\right]+K_{B}}\right)\left(1+\frac{\left[I\right]}{K_{I}}+\frac{\left[B\right]}{K_{B}}+\frac{\alpha_{I}\left[I\right][B]}{K_{I}K_{B}}\right)},$$

where Y is specific binding, *B*_max_ is the relative receptor expression, and *K_A_*, *K_B_*, and *K_I_* represent the equilibrium dissociation constants of [^3^H]DPCPX (A), the allosteric ligand (B), and NECA (I), respectively. The binding cooperativity between the allosteric ligand and [^3^H]DPCPX or NECA are denoted by α_A_ or α_I_, respectively. A cooperativity factor α > 1 describes positive cooperativity; a value 0 < α < 1 describes negative cooperativity, and a value of α = 1 describes neutral cooperativity.

NECA concentration–response curves from cAMP accumulation assays in the absence or presence of test compounds (30 μM) were fitted to the following three-parameter Hill equation to derive potency estimates:

[4] $$\mathrm{Response}=\mathrm{Basal}+\frac{\left(E_{\text{max}}-\mathrm{Basal}\right)\times \left[A\right]}{{\text{EC}}_{50}+\left[A\right]},$$

where EC_50_ is the concentration of NECA (A) in the absence or presence of VLS compounds that gives the midpoint response between basal and maximal effect (*E*_max_), which are the lower and upper asymptotes of the response, respectively.

Concentration–response curves for the interaction between NECA and allosteric ligands (3 to 30 μM), in a cAMP accumulation assay were globally fitted to the following operational model of allosterism (54).


[5] $$E=\frac{E_{m}{\left(\tau_{A}\left[A\right]\left(K_{B}+\alpha \beta \left[B\right]\right)+\tau_{B}\left[B\right]K_{A}\right)}^{n}}{{\left(\left[A\right]K_{B}+K_{A}K_{B}+\left[B\right]K_{A}+\alpha \left[A\right]\left[B\right]\right)}^{n}+{\left(\tau_{A}\left[A\right]\left(K_{B}+\alpha \beta \left[B\right]\right)+\tau_{B}\left[B\right]K_{A}\right)}^{n}}$$

where *E*_m_ is the maximal cellular response, *K_A_* and *K_B_* are the equilibrium dissociation constants of NECA (A) and the allosteric ligand (B), respectively, *τ*_A_ and *τ*_B_ are operational measures of NECA and allosteric ligand efficacy, respectively, *α* is the binding cooperativity factor, and *β* denotes the magnitude of the allosteric effect on orthosteric agonist efficacy, and *n* is the slope of the transducer function that links occupancy to the response. Orthosteric agonist and allosteric ligand affinity were constrained to values determined from radioligand binding.

## Supplementary Material

Appendix 01 (PDF)

Appendix 02 (PDF)

Appendix 03 (PDF)

## Data Availability

All study data are included in the article and/or supporting information.
