# Supplementary material for: Structure-based discovery of positive allosteric modulators of the A1 adenosine receptor
Source: Proc Natl Acad Sci U S A. 2025 Jul 7;122(28):e2421687122. doi: 10.1073/pnas.2421687122 (PMC12280925; doi:10.1073/pnas.2421687122)

PNAS

Supporting Information for

Structure-based discovery of positive allosteric modulators of the A_1 adenosine receptor.

Anh T.N. Nguyen[a,1], Nicolas Panel[b,1], Duc Duy Vo[c], Bui San Thai[a], Ling Yeong Chia[a], Cam Sinh Lu[a], Shane D. Hellyer[a], Monica Langiu[a], Manuela Jörg[d], Karen J. Gregory[a,e], Jan Kihlberg[c], Paul J. White[a], Peter J. Scammells[d], Arthur Christopoulos[a], Jens Carlsson[b,*], Lauren T. May[a,*]

*Lauren T. May and Jens Carlsson.
Email: lauren.may@monash.edu, or jens.carlsson@icm.uu.se

This PDF file includes:
Supporting text

Supporting Information Text

General synthetic procedures for 50-56: Materials and Methods

All reagents were purchased from Fluorochem, Sigma-Aldrich, Enamine and Chemtronica. DCM, methanol, DMF, and acetonitrile (99.9%) were purchased from VWR International AB, whereas THF was purchased from Sigma-Aldrich. Reagents and solvents were used as such without further purification. All reactions involving air or moisture-sensitive reagents or intermediates were performed under a nitrogen atmosphere. Mainly LC-MS was used for monitoring reactions using an Agilent 1100 series HPLC having a C18 Atlantis T3 column (3.0 × 50 mm, 5 μm). Acetonitrile–water (flow rate 0.75 mL/min over 6 min) was used as mobile phase and a Waters micromass Z_Q (model code: MM1) mass spectrometer with electrospray ionization mode was used for detection of molecular ions. TLC (Merck, silica gel 60 F_{254} plates) was sometimes used for monitoring reactions, particularly in the purification of compounds. Visualization of the developed TLC was done using UV light (254 nm) and staining with ninhydrin or anisaldehyde stain. After workup, organic phases were dried over Na_2SO_4/$MgSO_4$ and filtered before being concentrated under reduced pressure. ^1H and ^{13}C NMR spectra for the synthesized compounds were recorded at 298 K on an Agilent Technologies 400 MR spectrometer at 400 MHz or 100 MHz, respectively, or on Bruker Avance Neo spectrometers at 500/600 MHz or 125/150 MHz, respectively. Chemical shifts are reported in parts per million (ppm, δ) and referenced to the residual ^1H resonance of the solvent (CD_3OD δ 3.31) and the residual ^{13}C resonance of the solvent (CD_3OD δ 49.0). Splitting patterns are designated as follows: s (singlet), d (doublet), t (triplet), m (multiplet) and br (broad). Coupling constants (J) are listed in hertz (Hz). Preparative reversed-phase HPLC was performed on a Kromasil C8 column (250 × 21.2 mm, 5 μm) on a Gilson HPLC equipped with a Gilson 322 pump, a UV/Visible-156 detector and a 202 collector using acetonitrile-water gradients as eluents with a flow rate of 15 mL/min and detection at 210 or 254 nm. All tested compounds were purified by HPLC. ^1H NMR spectroscopy (600 MHz) and LCMS was used to determine the purity of the tested compounds, which all had a purity >95%.

General synthetic procedures for compounds **50-56**:

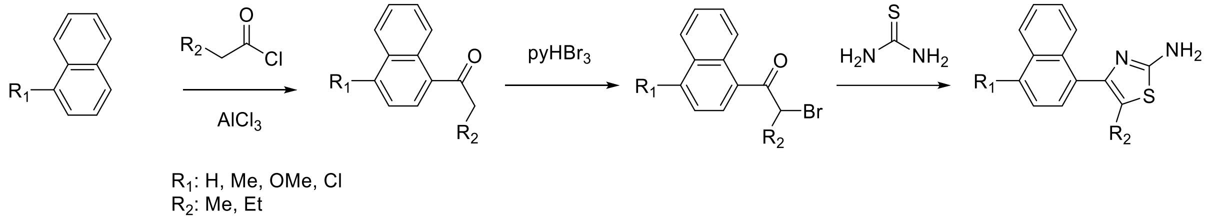

Briefly, compounds **50-56** were synthesized starting from naphthalene derivatives. Friedel-Craft aromatic acylation with acyl chlorides catalysed by $AlCl_3$ afforded ketone intermediates. Then, selective alpha bromination using pyridinium hydro tribromide afforded alpha bromo ketone intermediates which were condensed with thiourea to afford the target products **50-56**. All compounds were purified by HPLC.

Step 1: Acyl chloride (1.2 equiv.) was added to a stirred mixture of $AlCl_3$ (2 mmol, 2 equiv.) in DCM (10 mL) at 0°C. The mixture was stirred at 0°C for 30 min before adding naphthalene derivatives (1 equiv.) and then stirred overnight at rt. The reaction was quenched by adding cold H_2O (10 mL). The organic phase was separated and washed with saturated $NaHCO_3$ solution and brine, dried over Na_2SO_4 and concentrated under reduced pressure. The residue was purified by biotage (silica, 12 g) using a gradient of 10-20% ethyl acetate in hexane to afford the desired product. Yield: 47-76%.

Step 2+3: Pyridinium hydro tribromide (1 equiv.) was added to a stirred solution of ketone intermediate from step 1 (0.1 mmol, 1 equiv.) in a 1:1 mixture of $CHCl_3$:ACN (2 mL) at 0 °C. The solution was stirred overnight at rt. The solvents were removed, then thiourea (1.5-2 equiv.) and EtOH (1 mL) were added to the reaction mixture. The reaction was heated at 80 °C for 30 min. After cooling down, the solvent was removed and the residue was purified by hplc using a gradient of 20-100% ACN in H_2O (H_2O + 0.1% TFA) to afford the desired products as solid white TFA salts. Yield: 12-26 mg (31-65%, 2 steps).

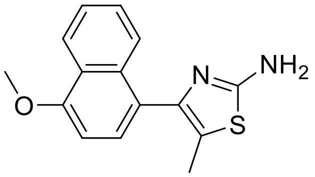

4-(4-methoxynaphthalen-1-yl)-5-methylthiazol-2-amine (**50**)
^{1}H NMR (600 MHz, CD_3OD) δ 8.35 (d, J = 8.3 Hz, 1H), 7.66 – 7.50 (m, 4H), 7.05 (d, J = 7.9 Hz, 1H), 4.08 (d, J = 1.2 Hz, 3H), 2.11 (d, J = 1.3 Hz, 3H).
^{13}C NMR (150 MHz, CD_3OD) δ 170.1, 158.7, 134.8, 134.1, 131.1, 128.9, 127.1, 126.9, 125.3, 123.7, 118.8, 118.6, 104.5, 56.4, 11.6.
LCMS (ESI+): calculated for $C_{15}H_{15}N_2OS$ $(M+H)^+$: 271.1; found 271.2.

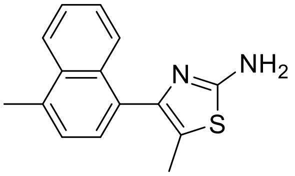

5-methyl-4-(4-methylnaphthalen-1-yl)thiazol-2-amine (**51**)
^{1}H NMR (600 MHz, CD_3OD) δ 8.19 – 8.15 (m, 1H), 7.74 – 7.69 (m, 1H), 7.67 – 7.57 (m, 2H), 7.48 (s, 2H), 2.77 (s, 3H), 2.10 (s, 3H).
^{13}C NMR (150 MHz, CD_3OD) δ 170.2, 138.9, 134.7, 134.2, 133.1, 130.0, 128.2, 127.7, 127.1, 126.1, 126.0, 125.2, 118.7, 19.7, 11.6.
LCMS (ESI+): calculated for $C_{15}H_{15}N_2S$ $(M+H)^+$: 255.1; found 255.2.

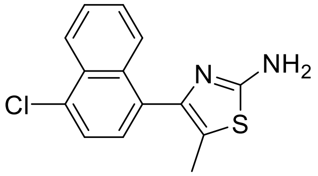

4-(4-chloronaphthalen-1-yl)-5-methylthiazol-2-amine (**52**)
^{1}H NMR (600 MHz, CD_3OD) δ 8.40 (dt, J = 8.6, 1.0 Hz, 1H), 7.82 – 7.68 (m, 4H), 7.57 (d, J = 7.6 Hz, 1H), 2.11 (s, 3H).
^{13}C NMR (150 MHz, CD_3OD) δ 170.4, 135.6, 134.3, 133.8, 132.2, 130.3, 129.5, 129.1, 126.9, 126.8, 126.4, 126.0, 119.6, 11.6.
LCMS (ESI+): calculated for $C_{14}H_{12}ClN_2S$ $(M+H)^+$: 275.0; found 275.1.

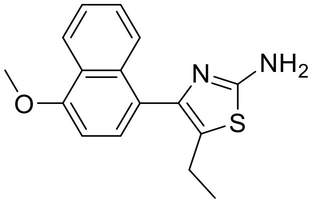

5-ethyl-4-(4-methoxynaphthalen-1-yl)thiazol-2-amine (**53**)
^{1}H NMR (600 MHz, CD_3OD) δ 8.37 – 8.32 (m, 1H), 7.65 – 7.53 (m, 3H), 7.51 (d, J = 7.9 Hz, 1H), 7.04 (d, J = 7.9 Hz, 1H), 4.08 (s, 3H), 2.46 (t, J = 7.4 Hz, 2H), 1.55 (h, J = 7.4 Hz, 2H), 0.84 (t, J = 7.4 Hz, 3H).
^{13}C NMR (150 MHz, CD_3OD) δ 170.0, 158.7, 134.7, 134.3, 131.1, 128.9, 127.1, 127.0, 125.3, 124.3, 123.7, 119.0, 104.5, 56.4, 29.1, 24.9, 13.7.
LCMS (ESI+): calculated for $C_{16}H_{17}N_2OS$ $(M+H)^+$: 288.1; found 288.2.

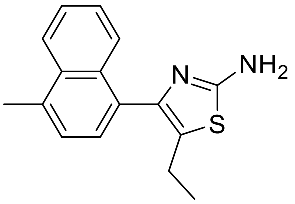

5-ethyl-4-(4-methylnaphthalen-1-yl)thiazol-2-amine (**54**)
^{1}H NMR (600 MHz, CD_3OD) *δ* 8.17 (dd, *J* = 8.1, 1.3 Hz, 1H), 7.71 (dd, *J* = 8.3, 1.4 Hz, 1H), 7.67 – 7.57 (m, 2H), 7.47 (s, 2H), 2.77 (s, 3H), 2.45 (t, *J* = 7.5 Hz, 2H), 1.54 (h, *J* = 7.4 Hz, 2H), 0.83 (t, *J* = 7.4 Hz, 3H).
^{13}C NMR (150 MHz, CD_3OD) *δ* 170.1, 138.9, 134.8, 134.2, 133.3, 130.0, 128.1, 127.7, 127.1, 126.2, 126.0, 125.5, 124.3, 119.1, 29.1, 24.8, 19.7, 13.6.
LCMS (ESI+): calculated for $C_{16}H_{17}N_2S$ $(M+H)^+$: 269.1; found 269.2.

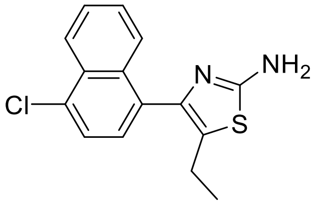

4-(4-chloronaphthalen-1-yl)-5-ethylthiazol-2-amine (**55**)
^{1}H NMR (600 MHz, CD_3OD) δ 8.40 (dt, *J* = 8.6, 1.0 Hz, 1H), 7.81 – 7.71 (m, 3H), 7.70 (ddd, *J* = 8.2, 6.8, 1.3 Hz, 1H), 7.55 (d, *J* = 7.6 Hz, 1H), 2.46 (t, *J* = 7.5 Hz, 2H), 1.55 (h, *J* = 7.4 Hz, 2H), 0.83 (t, *J* = 7.4 Hz, 3H).
^{13}C NMR (150 MHz, CD_3OD) δ 170.3, 135.5, 134.5, 134.0, 132.2, 130.3, 129.4, 129.1, 127.2, 126.9, 126.5, 126.0, 125.2, 29.1, 24.9, 13.6.
LCMS (ESI+): calculated for $C_{15}H_{14}ClN_2S$ $(M+H)^+$: 289.1; found 289.2.

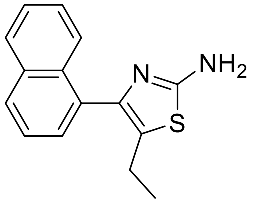

5-ethyl-4-(naphthalen-1-yl)thiazol-2-amine (**56**)
^{1}H NMR (600 MHz, CD_3OD) *δ* 8.09 (d, *J* = 8.1 Hz, 1H), 8.05 – 7.99 (m, 1H), 7.77 – 7.71 (m, 1H), 7.66 – 7.62 (m, 1H), 7.60 (ddd, *J* = 8.3, 6.7, 2.3 Hz, 3H), 2.48 (t, *J* = 7.5 Hz, 2H), 1.57 (h, *J* = 7.4 Hz, 2H), 0.85 (t, *J* = 7.4 Hz, 3H).
^{13}C NMR (150 MHz, CD_3OD) *δ* 170.1, 135.3, 135.1, 133.3, 131.7, 130.2, 129.8, 128.5, 127.8, 127.6, 126.4, 125.6, 124.6, 29.1, 24.9, 13.6.
LCMS (ESI+): calculated for $C_{15}H_{15}N_2S$ $(M+H)^+$: 255.1; found 255.2.

^{1}H NMR spectrum of **50** (600 MHz, CD_3OD)

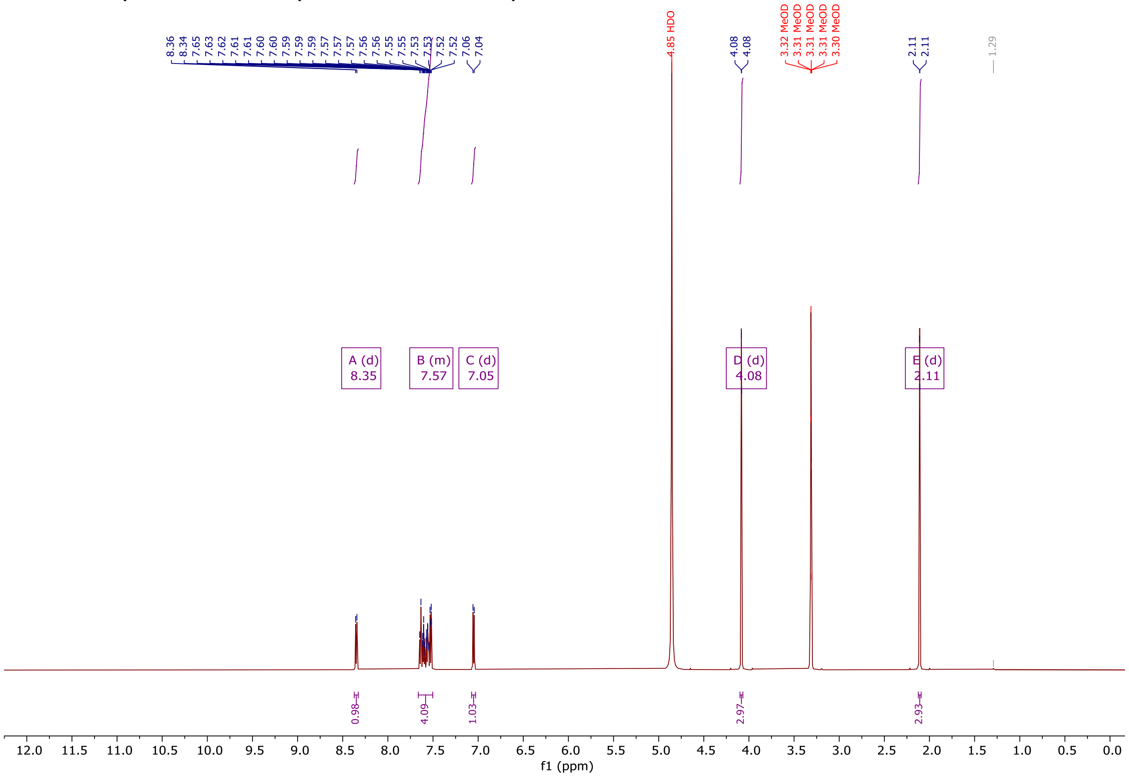

^{13}C NMR spectrum of **50** (600 MHz, CD_3OD)

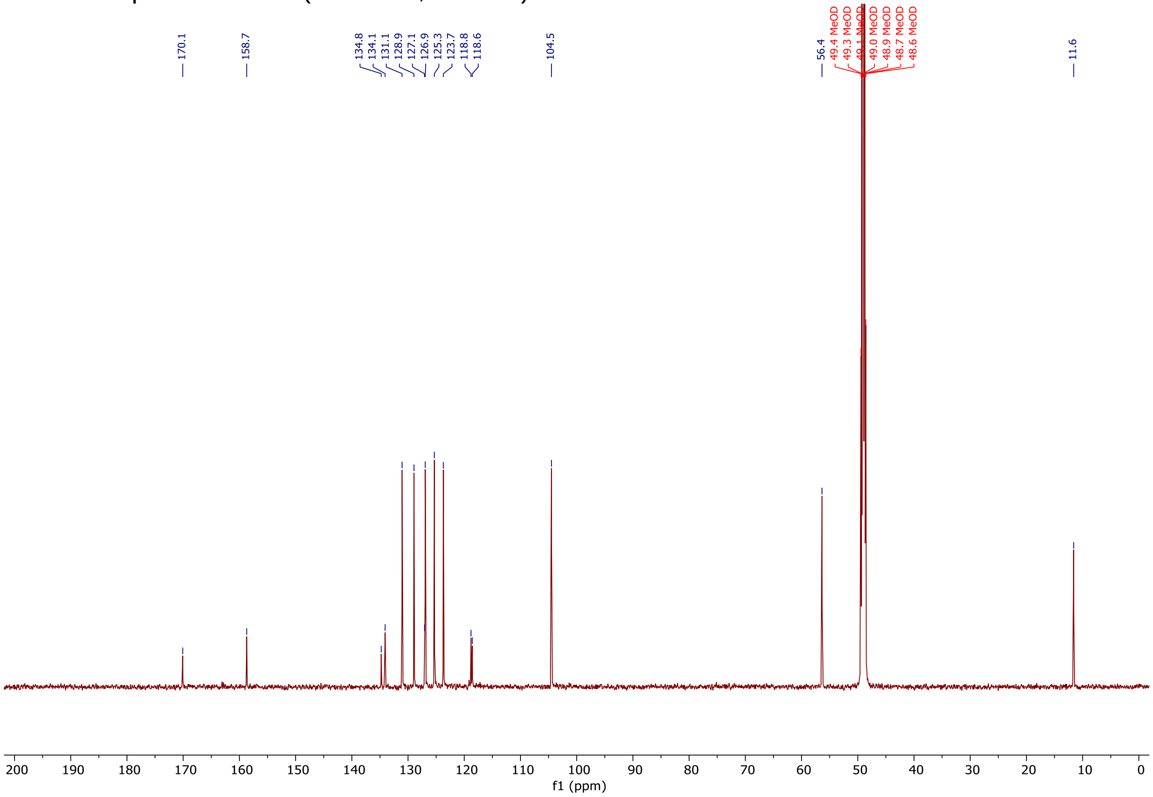

^{1}H NMR spectrum of **51** (600 MHz, CD_3OD)

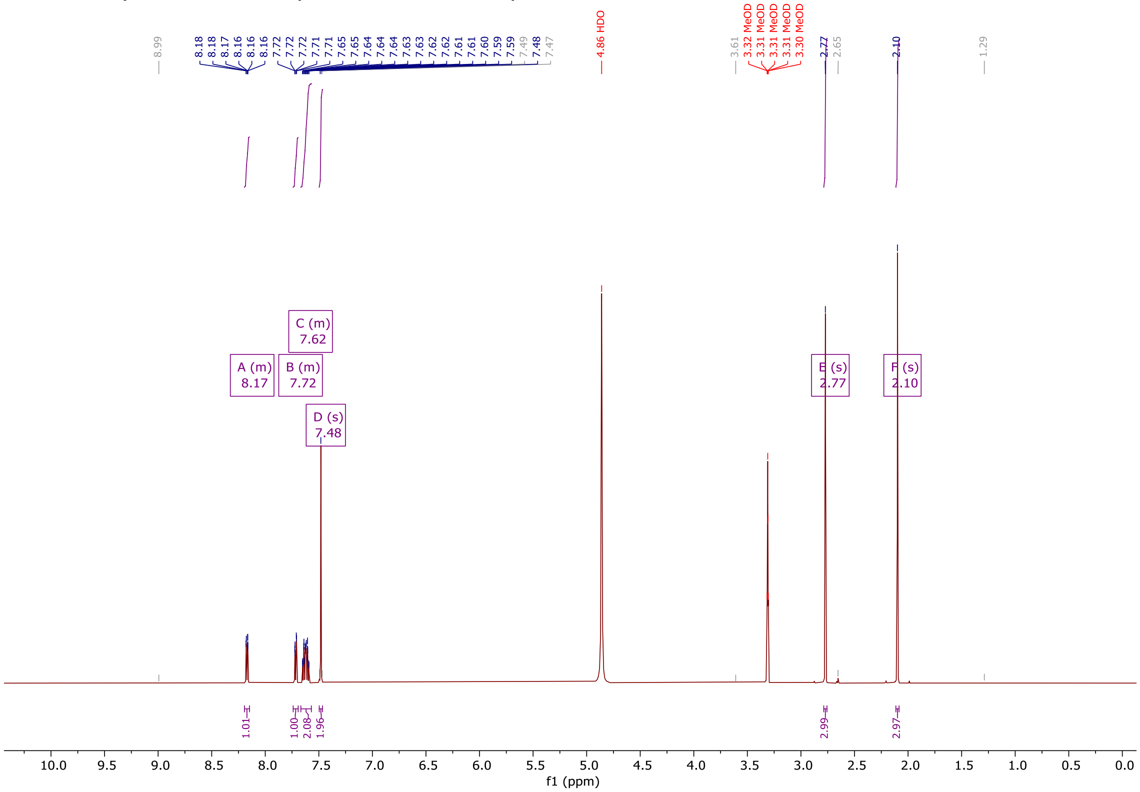

^{13}C NMR spectrum of **51** (600 MHz, CD_3OD)

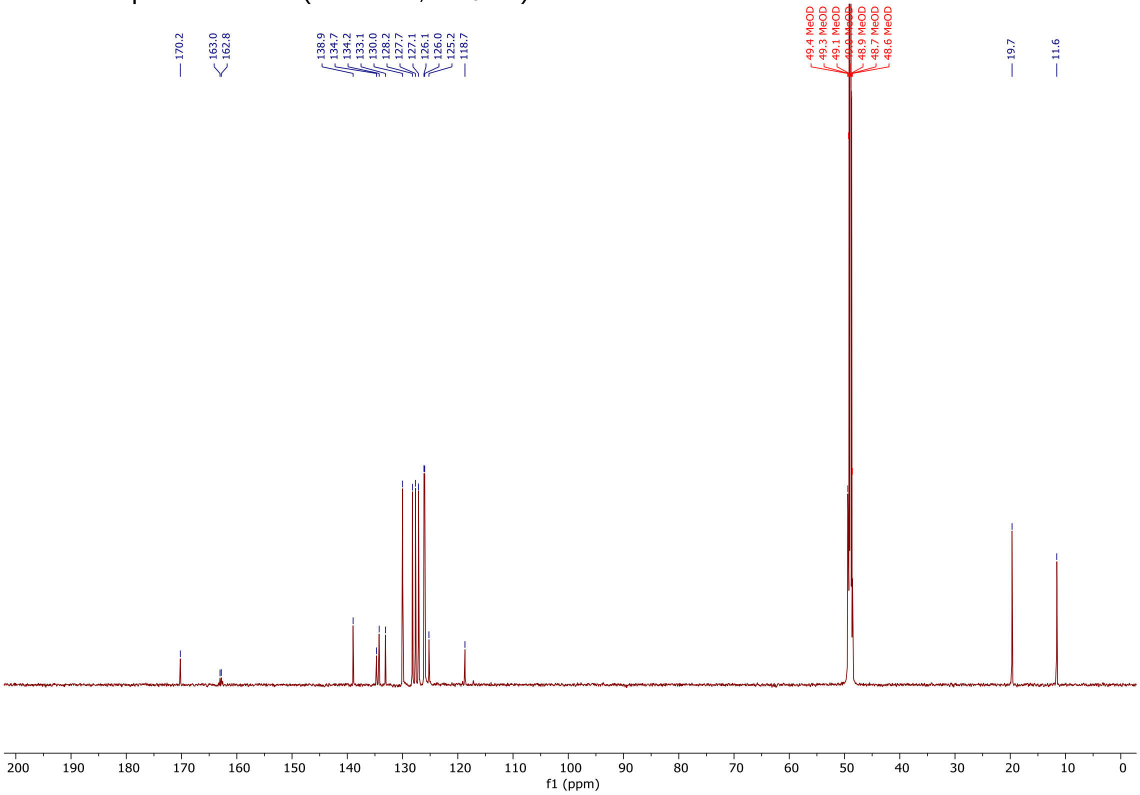

^{1}H NMR spectrum of **52** (600 MHz, CD_3OD)

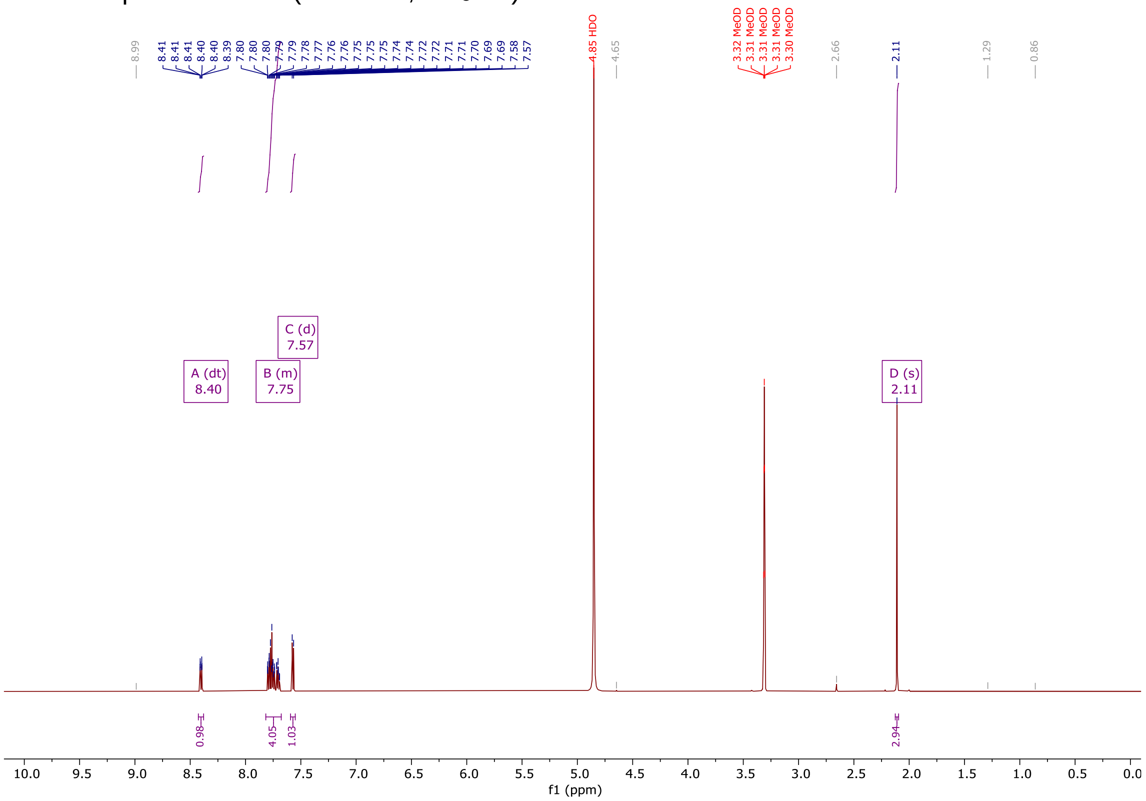

^{13}C NMR spectrum of **52** (600 MHz, CD_3OD)

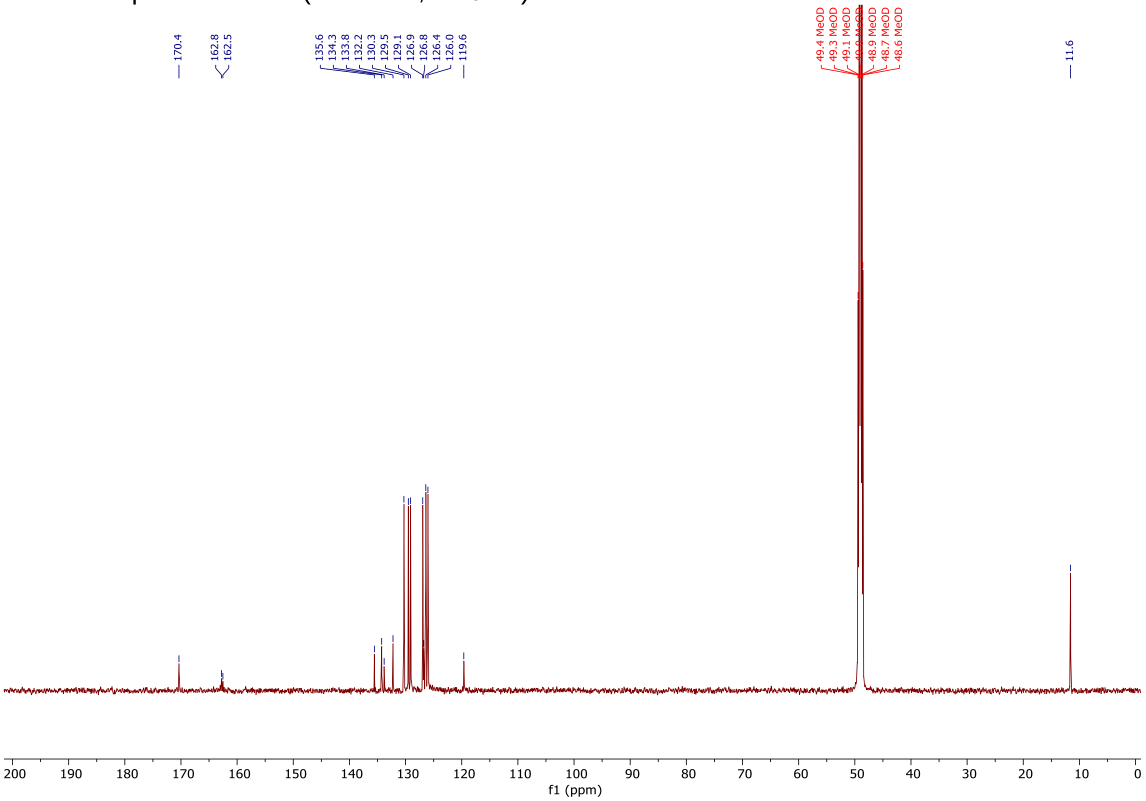

^{1}H NMR spectrum of **53** (600 MHz, CD_3OD)

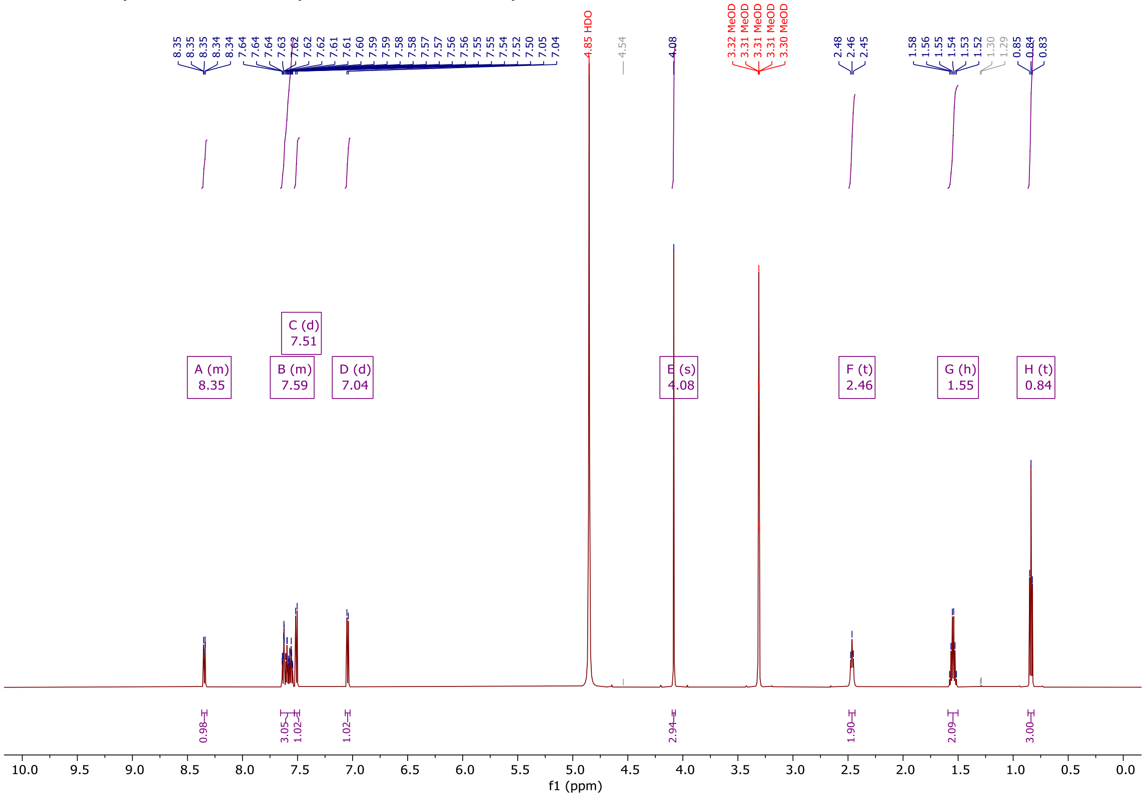

^{13}C NMR spectrum of **53** (600 MHz, CD_3OD)

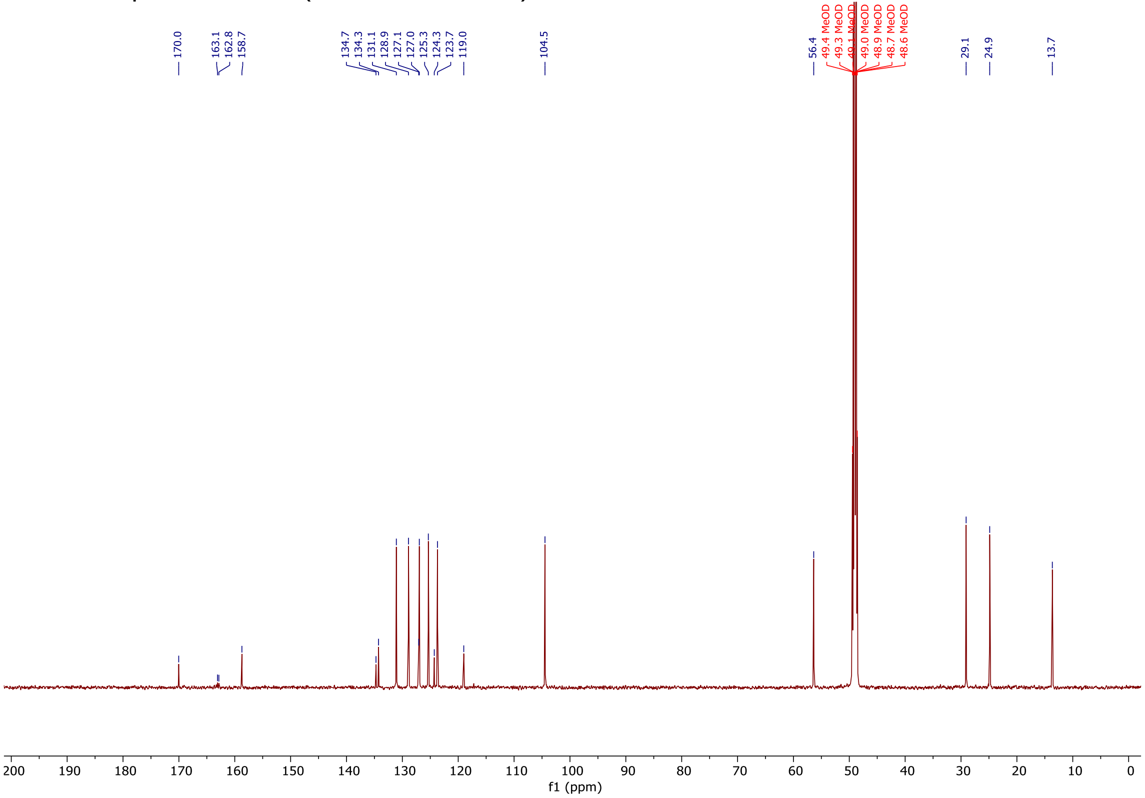

^{1}H NMR spectrum of **54** (600 MHz, CD_3OD)

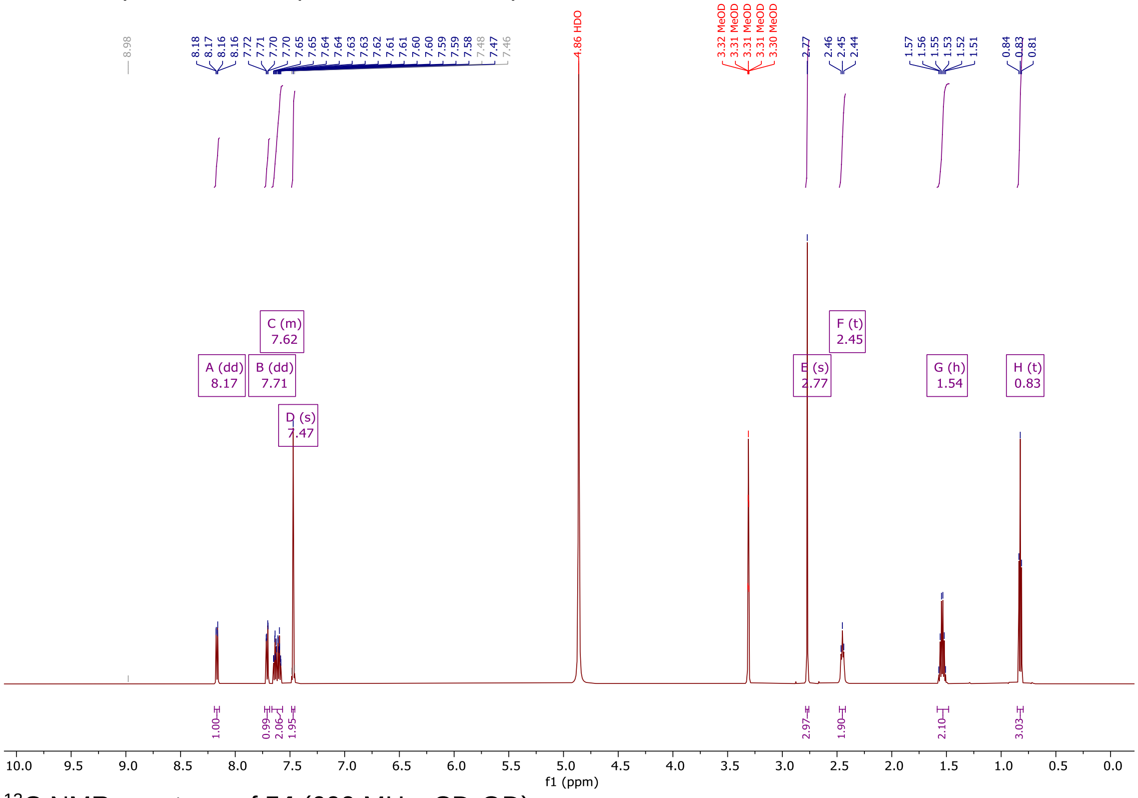

^{13}C NMR spectrum of **54** (600 MHz, CD_3OD)

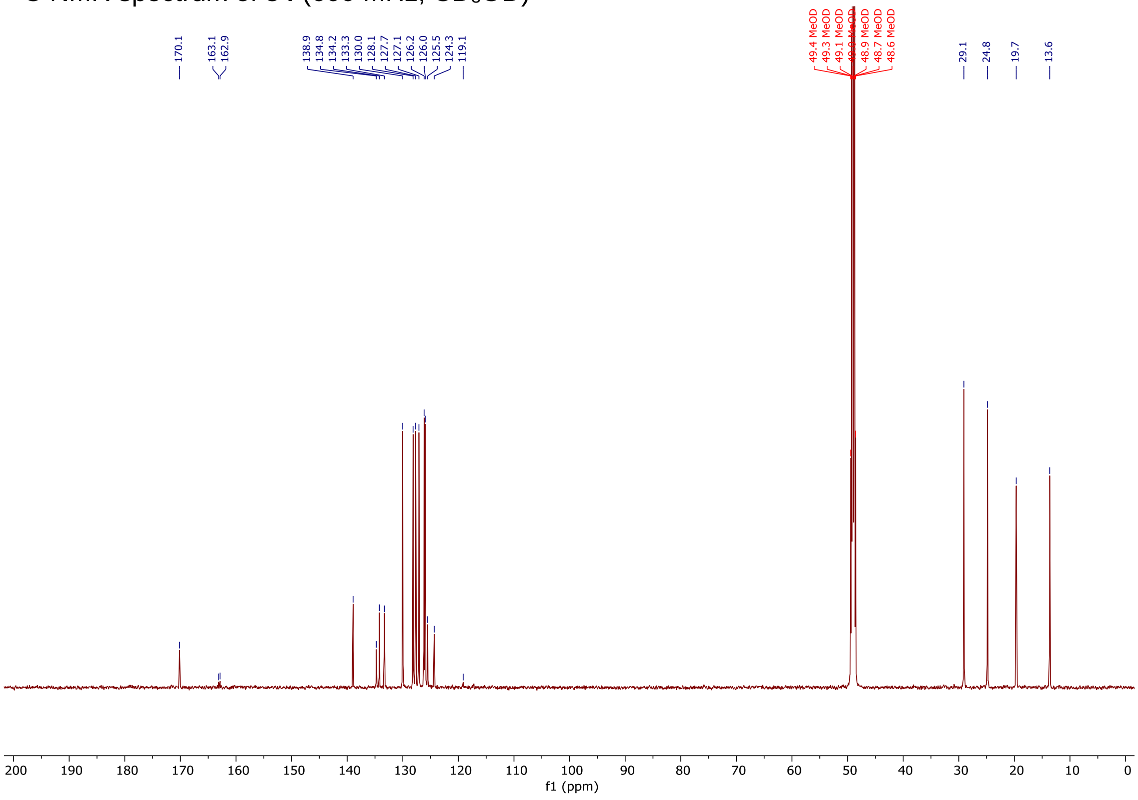

^{1}H NMR spectrum of **55** (600 MHz, CD_3OD)

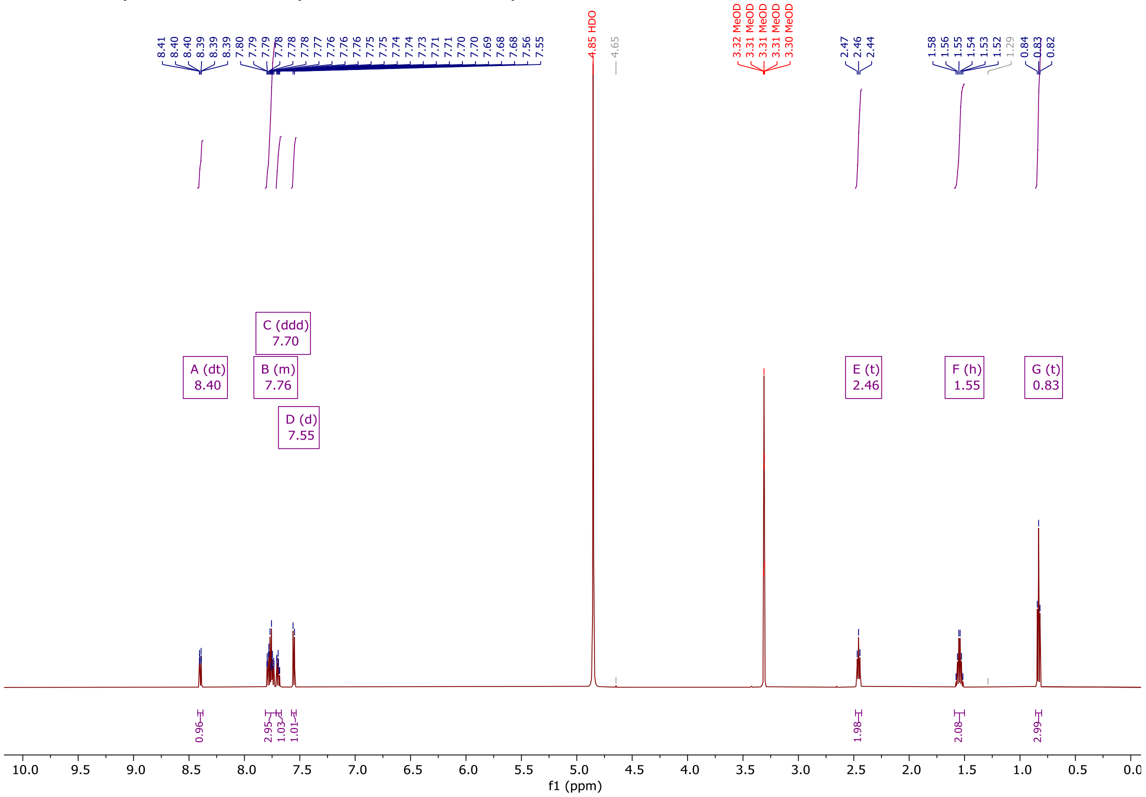

^{13}C NMR spectrum of **55** (600 MHz, CD_3OD)

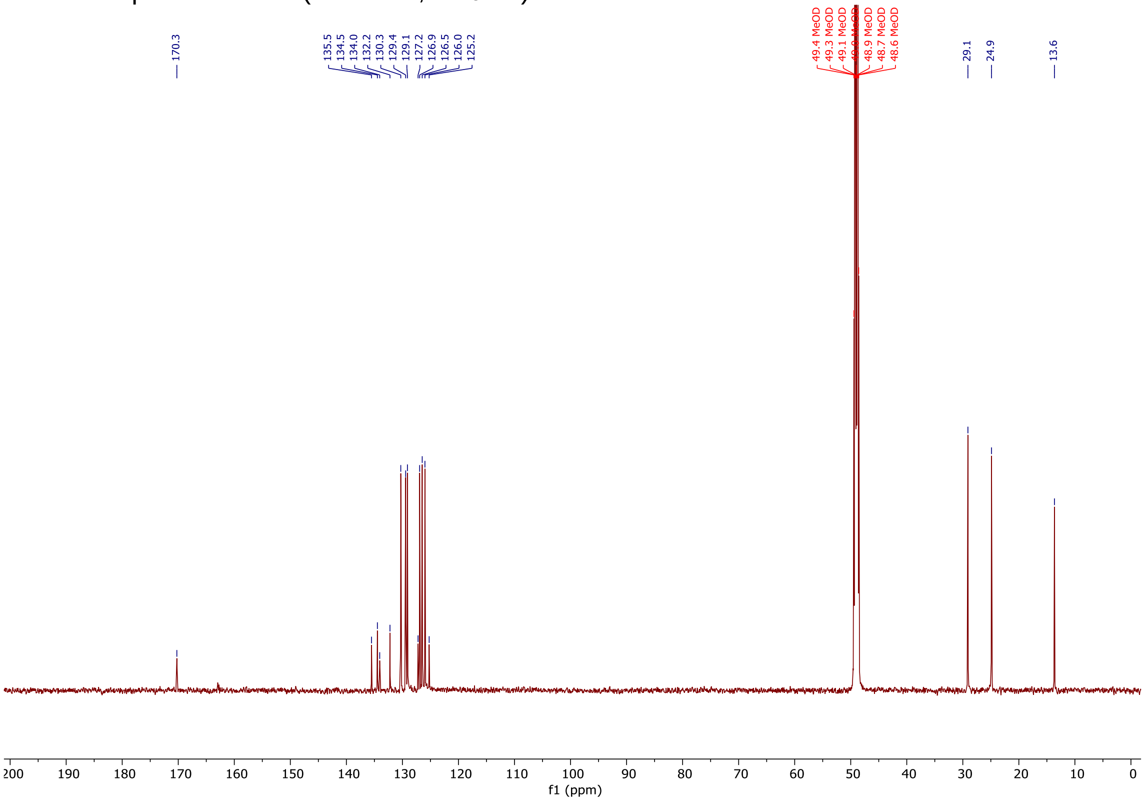

^{1}H NMR spectrum of **56** (600 MHz, CD_3OD)

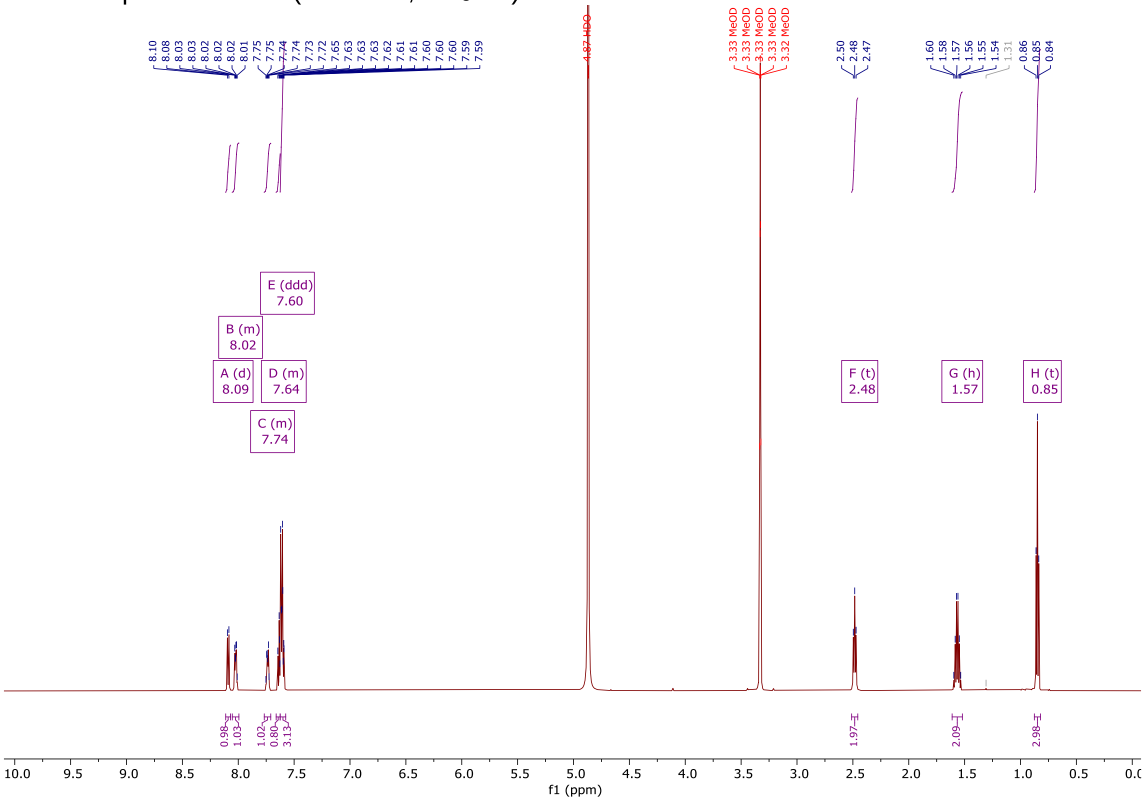

^{13}C NMR spectrum of **56** (600 MHz, CD_3OD)

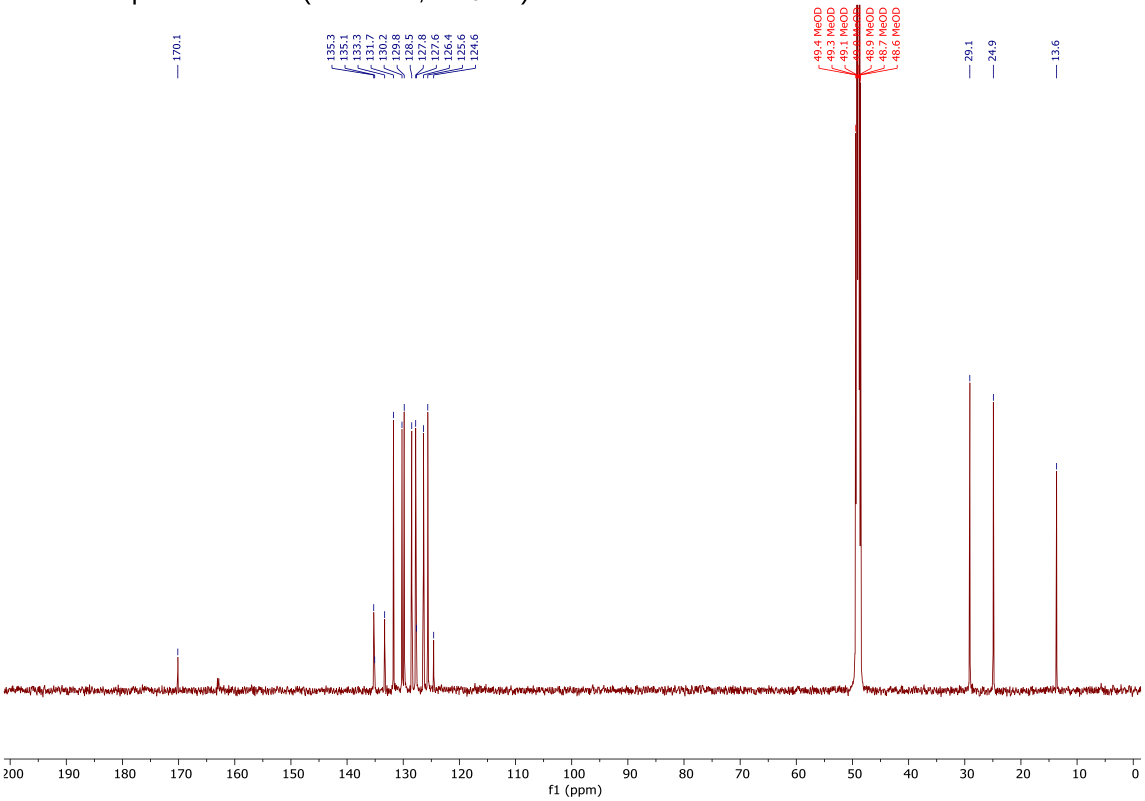

Supplement: Supplementary file 1 — Appendix 01 (PDF) [file pnas.2421687122.sapp01.pdf]
