# Supplementary material for: Structure-based discovery of positive allosteric modulators of the A1 adenosine receptor
Source: Proc Natl Acad Sci U S A. 2025 Jul 7;122(28):e2421687122. doi: 10.1073/pnas.2421687122 (PMC12280925; doi:10.1073/pnas.2421687122)
Supplement: Supplementary file 2 — Appendix 02 (PDF) [file pnas.2421687122.sapp02.pdf]

PNAS

Supporting Information for

Structure-based discovery of positive allosteric modulators of the A_1 adenosine receptor.

Anh T.N. Nguyen[a,1], Nicolas Panel[b,1], Duc Duy Vo[c], Bui San Thai[a], Ling Yeong Chia[a], Cam Sinh Lu[a], Shane D. Hellyer[a], Monica Langiu[a], Manuela Jörg[d], Karen J. Gregory[a,e], Jan Kihlberg[c], Paul J. White[a], Peter J. Scammells[d], Arthur Christopoulos[a], Jens Carlsson[b,*], Lauren T. May[a,*]

*Lauren T. May and Jens Carlsson.
Email**:** lauren.may@monash.edu, or jens.carlsson@icm.uu.se

This file includes:
Tables S1 to S3

Table S1. The structure and pharmacology of ligands identified from the first round of virtual screening. Pharmacological evaluation determined the influence of 30 µM compound on NECA affinity (pK_I) and potency (pEC_{50}).

ID	ZINC ID	2D structure	Lib.[a]	Rank #[b]	NECA pK_I ± compd[c]	NECA pEC_{50} ± compd[d]	Closest A_1R ligand structure from CHEMBL		
							Tc[e]	CHEMBL ID	2D structure
NECA					6.28 ± 0.07(3)	9.09 ± 0.24(6)			
**2**	ZINC19093895	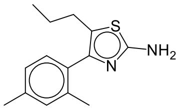	Frag	602	6.81 ± 0.04*(3)	9.7 ± 0.41(3)	0.43	CHEMBL38437	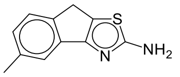
**3**	ZINC4234706	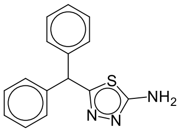	LL	17593	6.16 ± 0.05(3)	9.3 ± 0.37(3)	0.3	CHEMBL1373	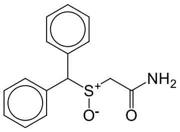
**4**	ZINC178947	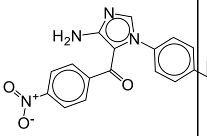	LL	665	6.36 ± 0.03(3)	9.49 ± 0.34(3)	0.46	CHEMBL549641	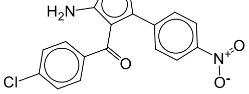
**5**	ZINC461252	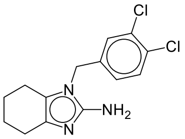	LL	4491	6.24 ± 0.07(3)	8.85 ± 0.29(3)	0.36	CHEMBL397821	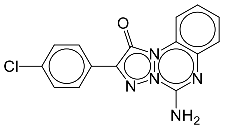
**6**	ZINC8580107	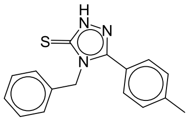	LL	22321	6.2 ± 0.08(3)	9.35 ± 0.34(3)	0.45	CHEMBL1099202	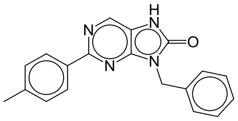
**7**	ZINC528405	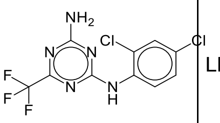	LL	6455	6.19 ± 0.06(3)	8.59 ± 0.44(3)	0.34	CHEMBL1712923	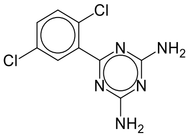
**8**	ZINC5589572	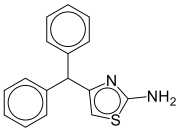	LL	23686	6.18 ± 0.06(3)	9.01 ± 0.50(3)	0.29	CHEMBL2377234	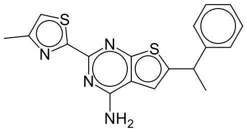
**9**	ZINC14629905	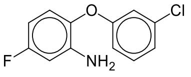	Frag	444	6.27 ± 0.06(3)	9.22 ± 0.51(3)	0.29	CHEMBL3735374	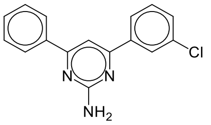
**10**	ZINC41638012	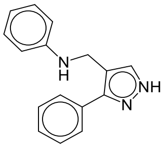	Frag	80	ND[f]	7.6 ± 0.32(3)	0.33	CHEMBL2146638	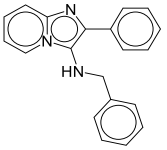
**11**	ZINC289970345	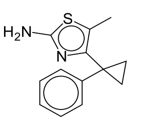	Frag	4700	6.35 ± 0.09(3)	9.51 ± 0.42(3)	0.4	CHEMBL2377236	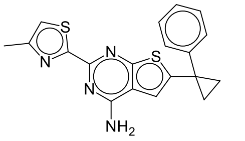

**12**	ZINC35886	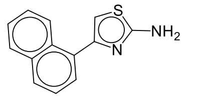	Frag	4541	7.2 ± 0.08* (3)	10.15 ± 0.65(3)	0.4	CHEMBL192577	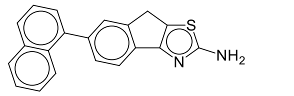
**13**	ZINC545662	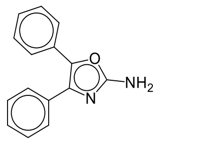	Frag	8856	5.4 ± 0.13* (3)	8.04 ± 0.36(3)	0.47	CHEMBL1071	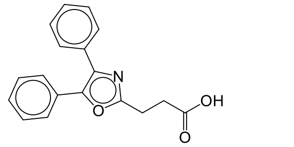
**14**	ZINC34955937	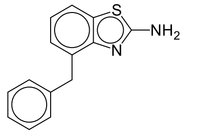	Frag	319	6.33 ± 0.02(3)	9.45 ± 0.48(3)	0.34	CHEMBL2322918	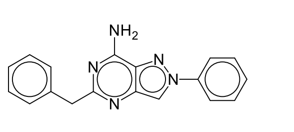
**15**	ZINC757569561	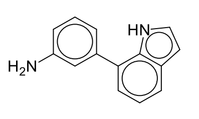	Frag	29536	6.09 ± 0.07(3)	9.68 ± 0.47(3)	0.33	CHEMBL323303	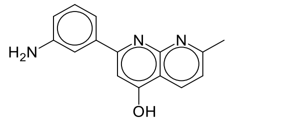
**16**	ZINC32256916	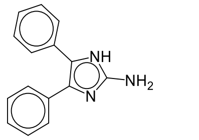	Frag	217	5.79 ± 0.14* (3)	8.55 ± 0.32(3)	0.44	CHEMBL1370747	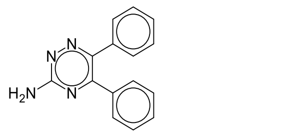
**17**	ZINC50227595	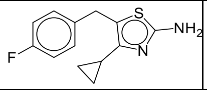	Frag	565	6.32 ± 0.02(3)	9.35 ± 0.46(3)	0.32	CHEMBL425000	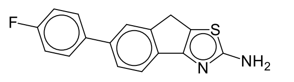
**18**	ZINC38235346	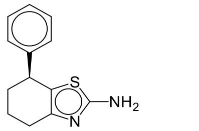	Frag	4750	6.17 ± 0.05(3)	9.27 ± 0.34(3)	0.42	CHEMBL289964	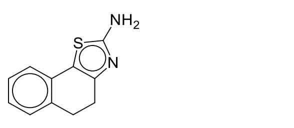
**19**	ZINC394726	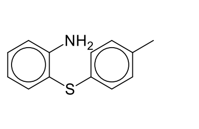	Frag	6835	6.81 ± 0.09* (3)	9.88 ± 0.54(3)	0.32	CHEMBL3930007	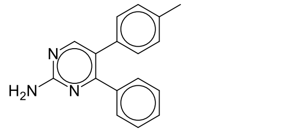
**20**	ZINC411284	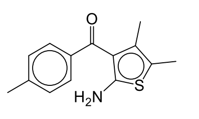	Frag	2097	7.22 ± 0.12* (3)	10.3 ± 0.29(3)	0.76	CHEMBL127147	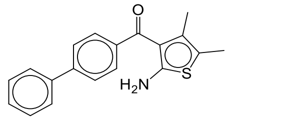
**21**	ZINC4218779	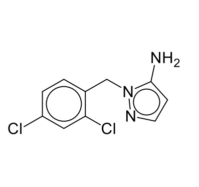	Frag	4200	6.2 ± 0.03(3)	9.33 ± 0.38(3)	0.33	CHEMBL257756	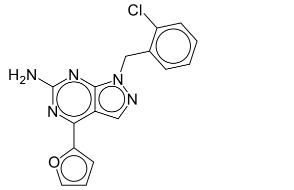
**22**	ZINC26439332	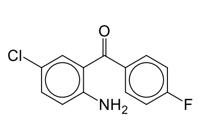	Frag	1684	6.79 ± 0.15* (3)	9.61 ± 0.47(3)	0.5	CHEMBL562466	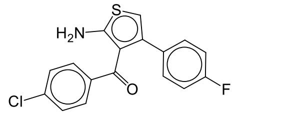
**23**	ZINC20081506	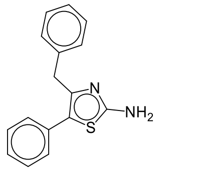	LL	66591	6.23 ± 0.08(3)	9.11 ± 0.38(3)	0.41	CHEMBL2377088	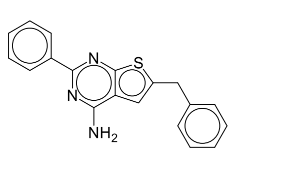

**24**	ZINC573061574	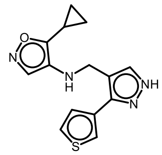	LL	28657	5.98 ± 0.11(3)	8.48 ± 0.36(3)	0.26	CHEMBL3786096	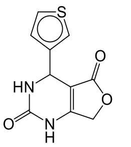
**25**	ZINC744871386	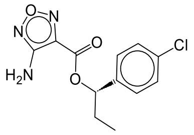	LL	10518	6.02 ± 0.07(3)	9.42 ± 0.35(3)	0.32	CHEMBL565	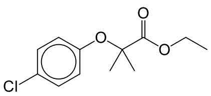
**26**	ZINC45885768	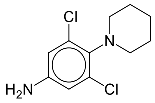	Frag	496	6.31 ± 0.07(3)	8.59 ± 0.45(3)	0.33	CHEMBL802	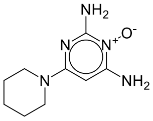
**27**	ZINC7097925	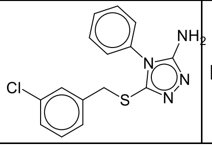	LL	21586	6.1 ± 0.12(3)	9.27 ± 0.44(3)	0.38	CHEMBL3317567	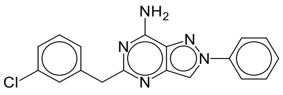

[a]Docked compounds were separated into two libraries based on compound molecular weights, i.e, fragment library (Frag, MW < 250 Da) and lead-like (LL, 250 < MW < 350 Da). [b]Docking rank of selected compounds from the A_1R screen from the two libraries. [c]NECA affinity (pK_I) determined from competition of 1 nM [^{3}H]DPCPX binding in the absence or presence of 30 μM compound in A_1R-FlpINCHO. Values represent the mean ± SEM; parentheses detailing the number of independent experiments performed in duplicate. [d]NECA potency (pEC_{50}) determined from cAMP accumulation assays in the absence or presence of 30 μM compound in A_1R-FlpINCHO cells. Values represent the mean ± SEM; parentheses detailing the number of independent experiments performed in duplicate. [e]Tanimoto coefficient for the most similar A_1R compounds in CHEMBL database using the ECFP4 fingerprint. [f]ND denotes not determined due to [^{3}H]DPCPX specific binding being virtually abolished in the presence of compound. *$p < 0.05$, one-way analysis of variance with Dunnett's post-test significant relative to NECA alone.

Table S2. The influence of extrahelical A_1R allosteric binding site mutations on novel PAM affinity (pK_B) and binding cooperativity ($\log\alpha$) with NECA at the A_1R.

		pK_B	$\log\alpha$
WT	**54**	5.25±0.13 (7)	0.92±016 (7)
	**56**	5.32±0.11 (7)	0.82±0.14 (7)
S246A	**54**	5.29±0.14 (11)	1.10±0.16 (11)
	**56**	5.51±0.16 (11)	0.93±0.18 (11)
G279A	**54**	5.67±0.13 (5)	-0.05±0.14* (5)
	**56**	5.52±0.28 (5)	-0.21±0.20* (5)

Values represent the mean ± SEM; parentheses detailing the number of independent experiments performed in duplicate. *$P < 0.05$, one-way ANOVA with Dunnett's post-hoc test compared to values determined from wild type (WT).

Table S3. List of selected compounds from the virtual screening and structure-guided optimization of A_1R PAMs.

ZINC ID	Cmpd#	SMILES	Vendor
ZINC19093895	**2**	CCCc1sc(N)nc1-c1ccc(C)cc1C	ChemBridge
ZINC4234706	**3**	NC1=NN=C(C(C2=CC=CC=C2)C2=CC=CC=C2)S1	ChemBridge
ZINC178947	**4**	NC1=C(C(=O)C2=CC=C([N+](=O)[O-])C=C2)N(C2=CC=C(Cl)C=C2)C=N1	ChemBridge
ZINC461252	**5**	NC1=[NH+]C2=CC=CC=C2N1CC1=CC=C(Cl)C(Cl)=C1	ChemBridge
ZINC8580107	**6**	CC1=CC=C(C2=NNC(=S)N2CC2=CC=CC=C2)C=C1	ChemBridge
ZINC528405	**7**	NC1=NC(NC2=CC=C(Cl)C=C2Cl)=NC(C(F)(F)F)=N1	ChemBridge
ZINC5589572	**8**	NC1=NC(C(C2=CC=CC=C2)C2=CC=CC=C2)=CS1	ChemBridge
ZINC14629905	**9**	NC1=CC(F)=CC=C1OC1=CC=CC(Cl)=C1	Enamine
ZINC41638012	**10**	C1=C(CNC2=CC=CC=C2)C(C2=CC=CC=C2)=NN1	Enamine
ZINC289970345	**11**	Cc1sc(N)nc1C1(c2ccccc2)CC1	Enamine
ZINC35886	**12**	NC1=NC(C2=C3C=CC=CC3=CC=C2)=CS1	Enamine
ZINC545662	**13**	NC1=NC(C2=CC=CC=C2)=C(C2=CC=CC=C2)O1	Enamine
ZINC34955937	**14**	NC1=NC2=C(CC3=CC=CC=C3)C=CC=C2S1	Enamine
ZINC757569561	**15**	NC1=CC=CC(C2=C3NC=CC3=CC=C2)=C1	Enamine
ZINC32256916	**16**	Nc1nc(-c2ccccc2)c(-c2ccccc2)[nH]1	Enamine
ZINC50227595	**17**	NC1=NC(C2CC2)=C(CC2=CC=C(F)C=C2)S1	Enamine
ZINC38235346	**18**	NC1=NC2=C(S1)[C@H](C1=CC=CC=C1)CCC2	Enamine
ZINC394726	**19**	Cc1ccc(Sc2ccccc2N)cc1	Enamine
ZINC411284	**20**	CC1=C(C)C(C(=O)C2=CC=C(C)C=C2)=C(N)S1	Enamine
ZINC4218779	**21**	Nc1ccnn1Cc1ccc(Cl)cc1Cl	Enamine
ZINC26439332	**22**	NC1=CC=C(Cl)C=C1C(=O)C1=CC=C(F)C=C1	Enamine
ZINC20081506	**23**	NC1=NC(CC2=CC=CC=C2)=C(C2=CC=CC=C2)S1	Enamine
ZINC573061574	**24**	c1cc(-c2n[nH]cc2CNc2cnoc2C2CC2)cs1	Enamine
ZINC744871386	**25**	CC[C@H](OC(=O)C1=NON=C1N)C1=CC=C(Cl)C=C1	Enamine
ZINC45885768	**26**	NC1=CC(Cl)=C(N2CCCCC2)C(Cl)=C1	Vitas-M
ZINC7097925	**27**	Nc1nnc(SCc2cccc(Cl)c2)n1-c1ccccc1	Vitas-M
ZINC2585761	**28**	CCCc1c(nc(s1)N)c2ccccc2	Fluorochem
ZINC453334	**29**	CCCc1c(nc(s1)N)c2ccc(cc2)Cl	Chembridge
ZINC1459986	**30**	CCCc1c(nc(s1)N)c2ccc(cc2)OC	Chembridge
ZINC304917	**31**	CCCc1c(nc(s1)N)c2ccc(cc2)C	Chembridge
ZINC305349	**32**	CCCc1c(nc(s1)N)c2ccc(c(c2)C)C	Chembridge
ZINC22289179	**33**	CCCc1c(nc(s1)N)c2ccc(c(c2)OC)OC	Chembridge
ZINC19093899	**34**	CCCc1c(nc(s1)N)c2cc(ccc2C)C	Chembridge
ZINC2545435	**35**	COc1ccc(c2c1cccc2)c3csc(n3)N	Enamine
ZINC245607	**36**	COc1ccc2ccccc2c1c3csc(n3)N	Fluorochem
ZINC278988	**37**	Cc1ccc(c2c1cccc2)c3csc(n3)N	Vitas-M
ZINC3817441	**38**	Cc1c(nc(s1)N)c2cccc3c2cccc3	Enamine
ZINC241326	**39**	Nc1sc(c(n1)c1ccccc1)C(C)(C)C	Chembridge
ZINC361138	**40**	Nc1nc(c(s1)c1ccccc1)c1ccccc1	Enamine
ZINC2506007	**41**	Nc1sc(c(n1)c1ccc(cc1)C(C)(C)C)C	Enamine
ZINC8429446	**42**	Nc1sc(c(n1)c1ccc2c(c1)CCC2)C	Enamine
ZINC1042315	**43**	FC(c1cc(cc(c1)C(F)(F)F)c1csc(n1)N)(F)F	Fluorochem
ZINC35850	**44**	Nc1scc(n1)c1ccc2c(c1)cccc2	Enamine
ZINC11535708	**45**	Nc1scc(n1)Cc1ccccc1	Chembridge
ZINC13005271	**46**	Nc1scc(n1)c1cn(c2c1cccc2)C	Chembridge
ZINC69857	**47**	Nc1scc(n1)c1csc2c1cccc2	Enamine
ZINC3092600	**48**	Nc1scc(n1)c1cc2c(o1)cccc2	Enamine
ZINC3888951	**49**	Nc1scc(n1)c1sc2c(c1C)cccc2	Enamine
DV923	**50**	COc1ccc(c2c(C)sc(N)n2)c2ccccc12	In-House
DV924	**51**	Cc1c(nc(N)s1)c1ccc(C)c2ccccc12	In-House
DV925	**52**	Cc1c(nc(N)s1)c1ccc(Cl)c2ccccc12	In-House
DV927	**53**	CCCc1c(nc(N)s1)c1ccc(OC)c2ccccc12	In-House
DV928	**54**	CCCc1c(nc(N)s1)c1ccc(C)c2ccccc12	In-House
DV929	**55**	CCCc1c(nc(N)s1)c1ccc(Cl)c2ccccc12	In-House
DV934	**56**	CCCc1c(nc(N)s1)c1cccc2ccccc12	In-House
