# Supplementary material for: Structure-based discovery of positive allosteric modulators of the A1 adenosine receptor
Source: Proc Natl Acad Sci U S A. 2025 Jul 7;122(28):e2421687122. doi: 10.1073/pnas.2421687122 (PMC12280925; doi:10.1073/pnas.2421687122)
Supplement: Supplementary file 3 — Appendix 03 (PDF) [file pnas.2421687122.sapp03.pdf]

PNAS

Supporting Information for

Structure-based discovery of positive allosteric modulators of the A_1 adenosine receptor.

Anh T.N. Nguyen[a,1], Nicolas Panel[b,1], Duc Duy Vo[c], Bui San Thai[a], Ling Yeong Chia[a], Cam Sinh Lu[a], Shane D. Hellyer[a], Monica Langiu[a], Manuela Jörg[d], Karen J. Gregory[a,e], Jan Kihlberg[c], Paul J. White[a], Peter J. Scammells[d], Arthur Christopoulos[a], Jens Carlsson[b,*], Lauren T. May[a,*]

*Lauren T. May and Jens Carlsson.
Email: lauren.may@monash.edu, or jens.carlsson@icm.uu.se

This file includes:
Figures S1 to S10

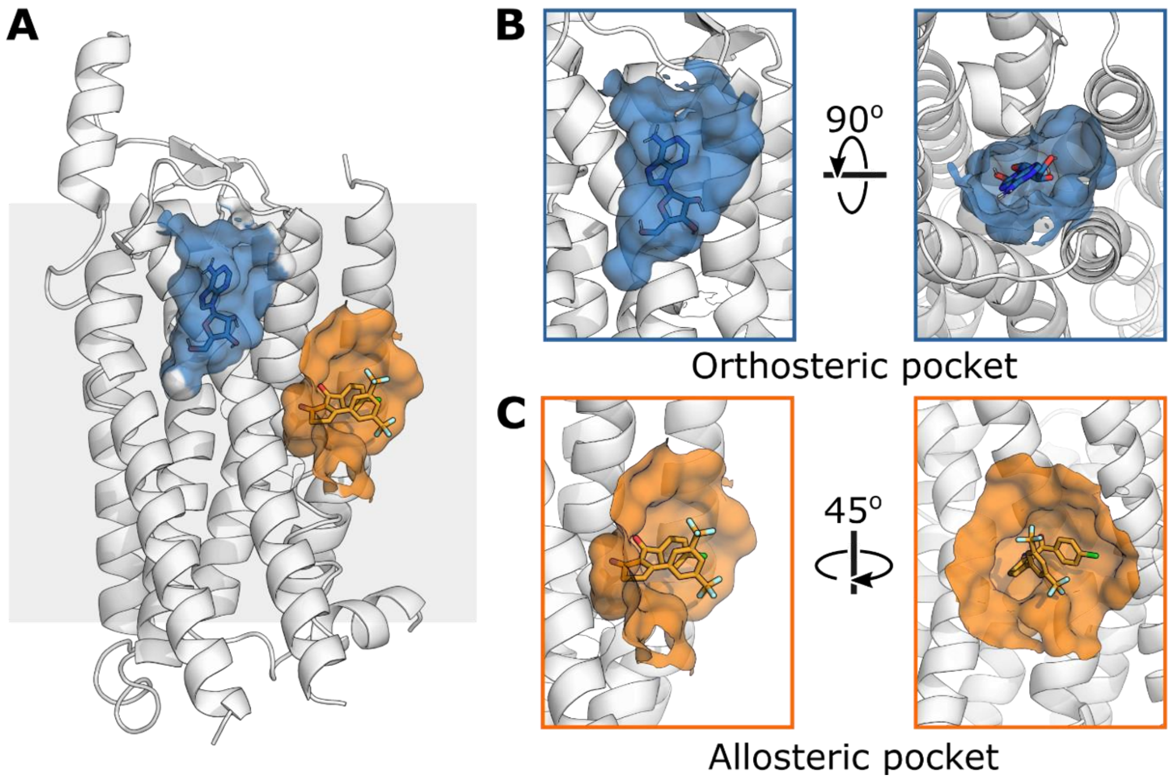

Fig. S1. The orthosteric and allosteric sites of the A_1R. (*A*) The locations of the orthosteric (blue surface) and allosteric (orange surface) sites are shown. The receptor is depicted as a white cartoon, and the ligands are represented as sticks. The location of the phospholipid bilayer is shown as a grey square. The orthosteric site is a deep, enclosed pocket (*B*), whereas the allosteric site is shallow and open (*C*).

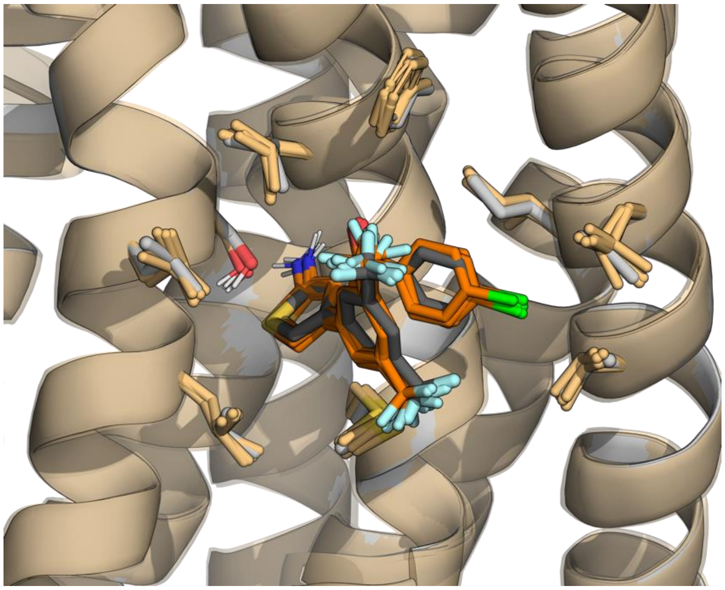

Fig. S2. MD simulation snapshots of the A_1R allosteric pocket. The five MD simulation snapshots used in the virtual screen were superimposed onto the cryo-EM structure of the A_1R-MIPS521 complex. The MD snapshots of the receptor are shown as a beige cartoon, with selected side chains (beige) and MIPS521 (orange) depicted as sticks. The receptor from the cryo-EM structure is shown as a white cartoon, with selected side chains (white) and MIPS521 (black) depicted as sticks.

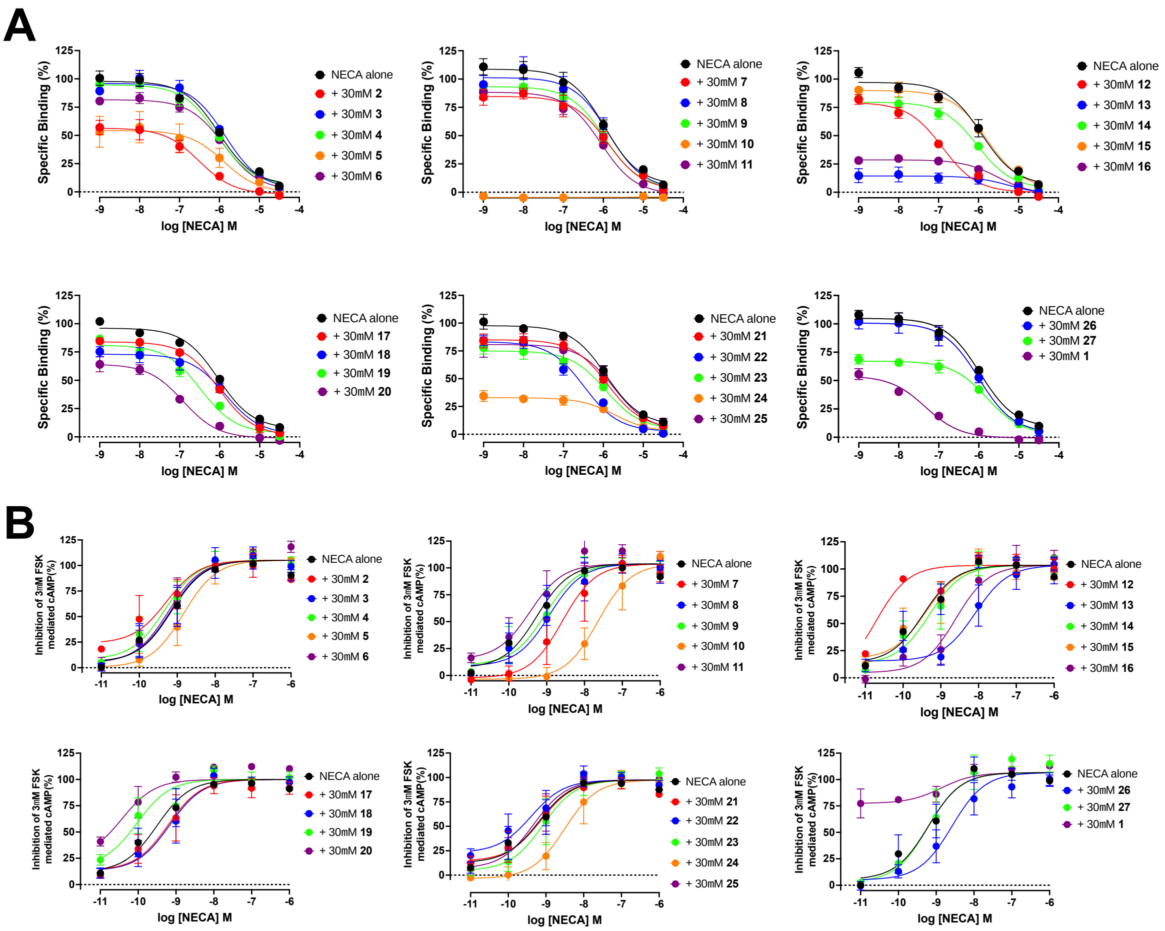

Fig. S3. Influence of the first round of virtual screening compounds on NECA binding and function at the A_1R. (*A*) NECA-mediated inhibition of 1 nM $[^3H]$DPCPX binding in the absence or presence of 30 μM **1** - **27** in A_1R-FlpInCHO cells. (*B*) Inhibition of 3 μM forskolin-stimulated cAMP accumulation mediated by NECA in the absence or presence of 30 μM **1** - **27** in A_1R-FlpInCHO cells. Data represent the mean ± SEM from n=3-4 individual replicates performed in duplicate. Error bars not shown lie within the dimensions of the symbol.

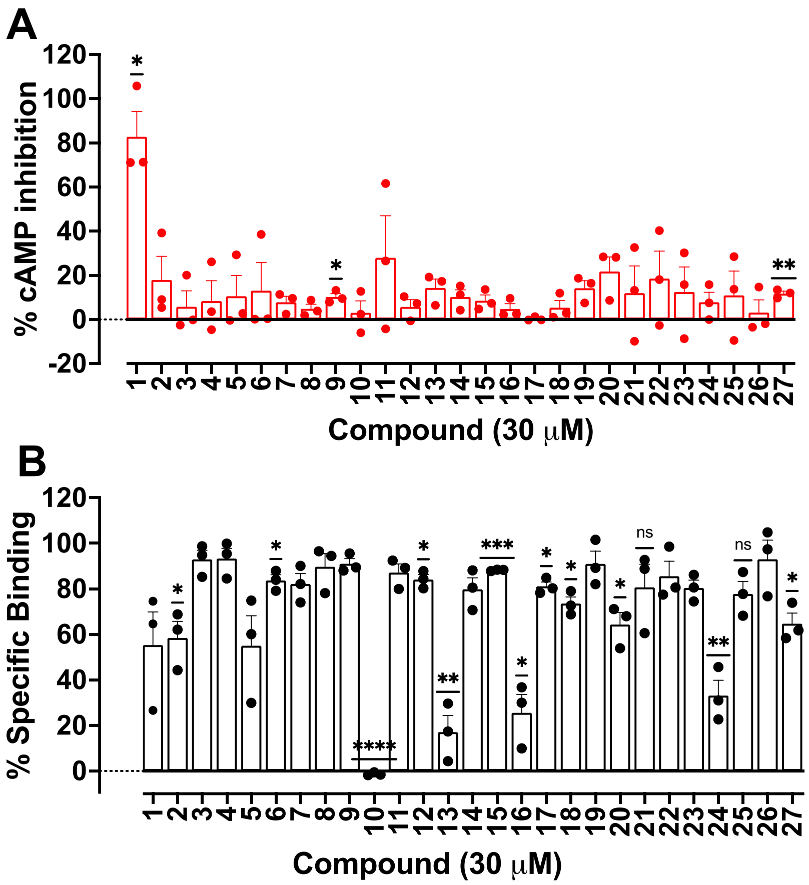

Fig. S4. Effect of virtual screening compounds at the A_1R in the absence of orthosteric agonist. (*A*) Influence of 30 μM **1-27** on 3 μM forskolin-stimulated cAMP accumulation in A_1R-FlpInCHO cells. (*B*) Influence 30 μM **1-27** on specific binding of 1nM [^{3}H]DPCPX in A_1R-FlpInCHO cells. Data represent the mean ± SEM from n=3-4 individual replicates performed in duplicate. *$p < 0.05$, **$p < 0.01$, ***$p<0.001$, ****$p<0.0001$ one-sample t-test relative to baseline.

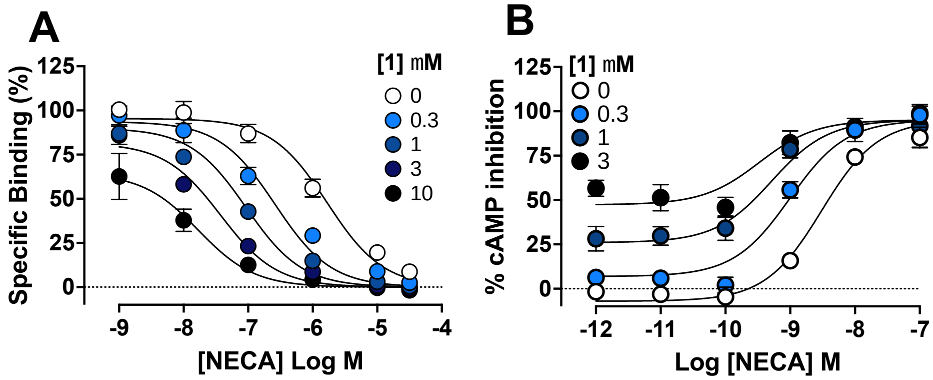

Fig. S5. Interaction profile of NECA and **1** (MIPS521) in [^{3}H]DPCPX competition binding (*A*) and inhibition of forskolin-stimulated cAMP accumulation (*B*) in A_1R-FlpInCHO cells. Error bars not shown lie within the dimensions of the symbol. Data represent the mean ± SEM from n=4 individual replicates performed in duplicate.

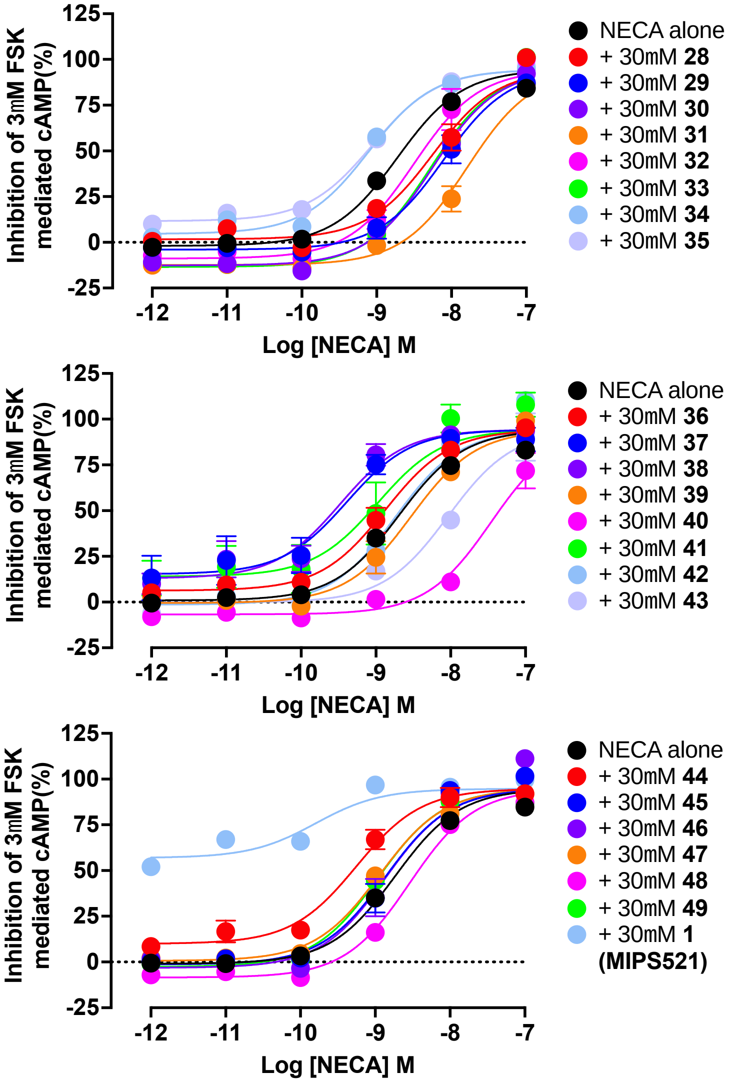

Fig. S6. Influence of second round of virtual screening compounds on NECA potency at the A_1R. Inhibition of 3 µM forskolin-stimulated cAMP accumulation mediated by NECA in the absence or presence of 30 µM **28** - **49** in A_1R-FlpInCHO cells. Data represent the mean ± SEM from n=3-4 individual replicates performed in duplicate. Error bars not shown lie within the dimensions of the symbol.

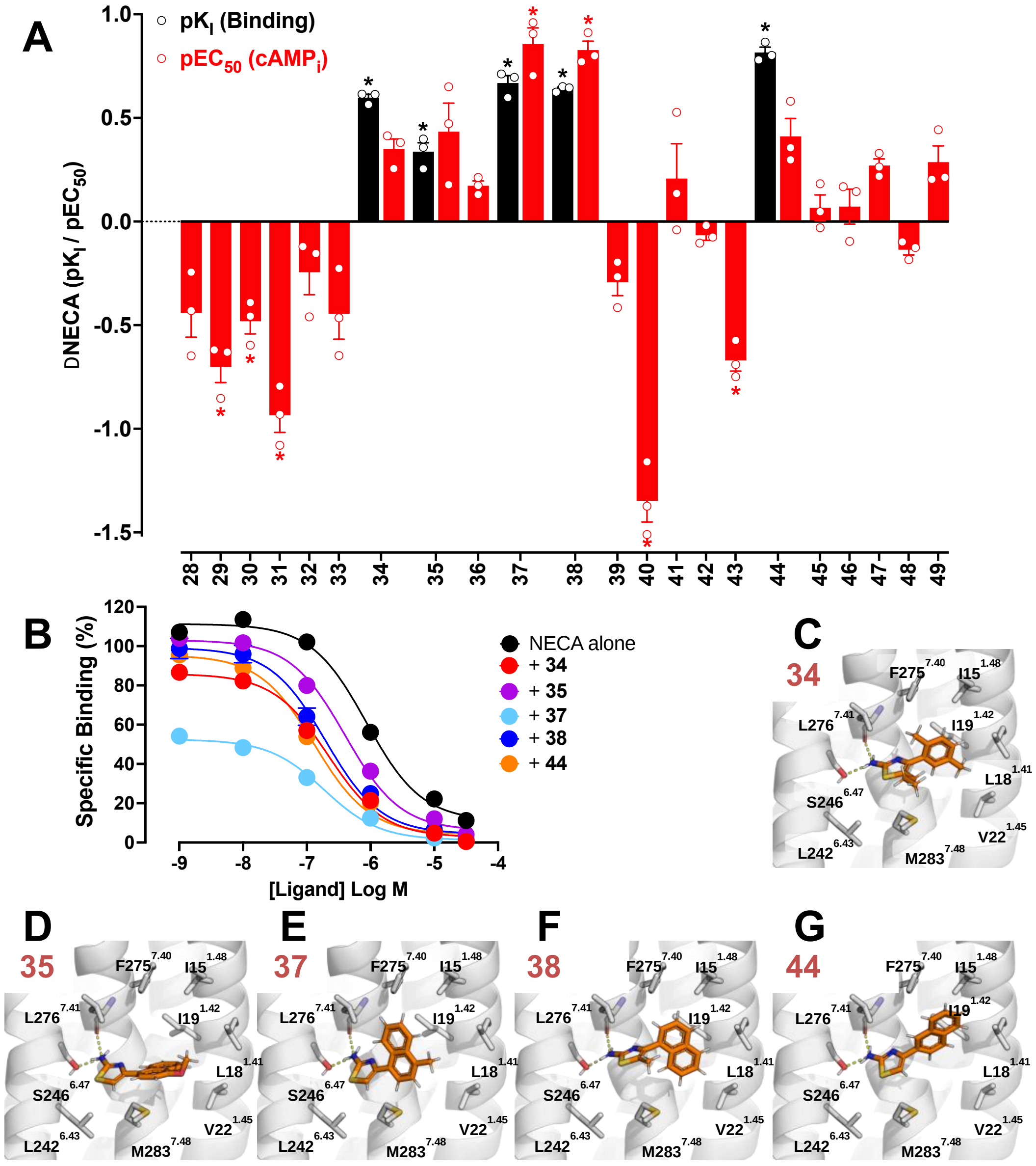

Fig. S7. Structure-guided optimization (second round) and pharmacological profiling of A_1R PAMs. (*A*) The change in NECA potency (pEC_{50}) and affinity (pK_I) in the presence of 30 μM **28** – **49** in A_1R-FlpInCHO cells using inhibition of cAMP accumulation ($cAMP_i$) and [^{3}H]DPCPX competition binding assays. *p< 0.05, one sample t-test compared to a hypothetical value of 0. (*B*) NECA concentration-response curves for the [^{3}H]DPCPX competition binding to A_1R-FlpInCHO cells in the absence and presence of 30 μM selected hit compounds. Data represent the mean ± SEM from n=3 individual replicates performed in duplicate. Error bars not shown lie within the dimensions of the symbol. (*C-G*) Predicted binding modes of compounds **34** (*C*), **35** (*D*), **37** (*E*), **38** (*F*), and **44** (*G*). The ligands are shown as sticks with carbon atoms in orange. The receptor is shown as a grey cartoon with key residues in sticks. Hydrogen bonds between the ligands and L276 and S246 are indicated with yellow dashed lines.

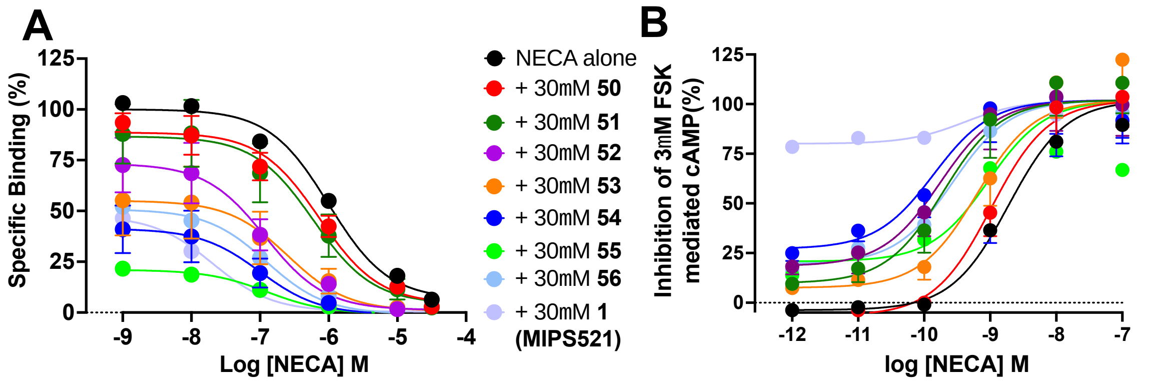

Fig. S8. Influence of the third round of virtual screening compounds on NECA affinity and potency at the A_1R. (*A*) NECA-mediated inhibition of 1 nM $[^3H]$DPCPX binding in the absence or presence of 30 μM **50 - 56** in A_1R-FlpInCHO cells. (*B*) Inhibition of 3 μM forskolin-stimulated cAMP accumulation mediated by NECA in the absence or presence of 30 μM **50 - 56** in A_1R-FlpInCHO cells. Data represent the mean ± SEM from n=3-4 individual replicates performed in duplicate. Error bars not shown lie within the dimensions of the symbol.

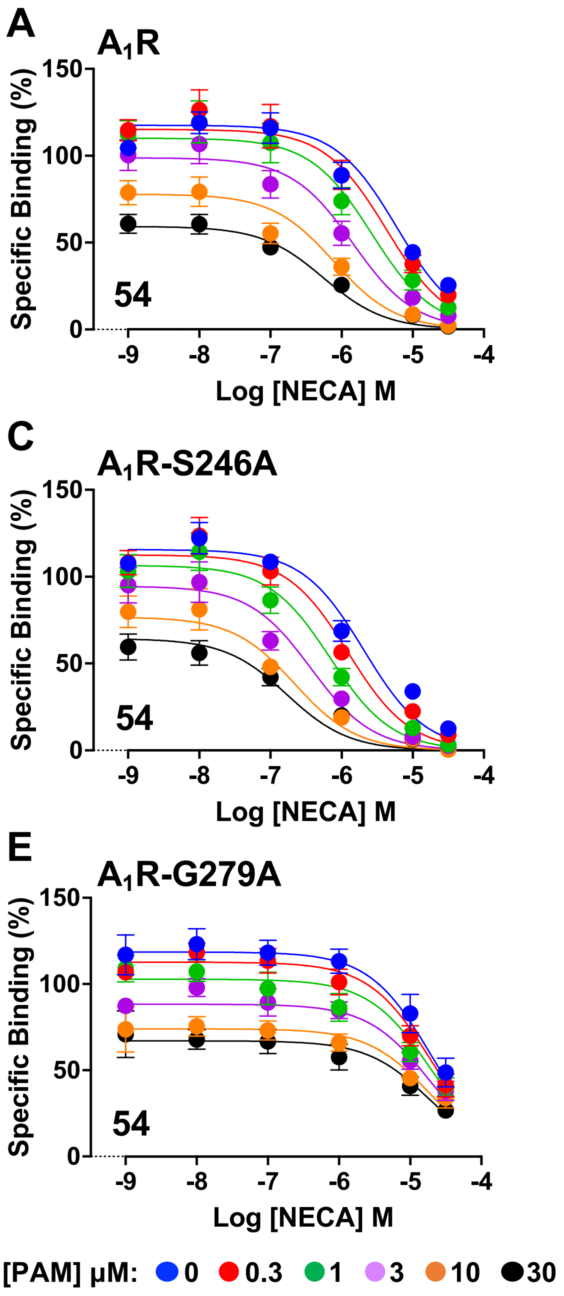

Fig. S9. [^{3}H]DPCPX interaction binding between NECA and A_1R PAMs, **54** and **56**, at the wild-type A_1R-FlpINCHO (*A-B*), S246A-A_1R-FlpINCHO (*C-D*) and G279A-A_1R-FlpINCHO (*E-F*). Data represent the mean ± SEM from n=3-4 individual replicates performed in duplicate. Error bars not shown lie within the dimensions of the symbol.

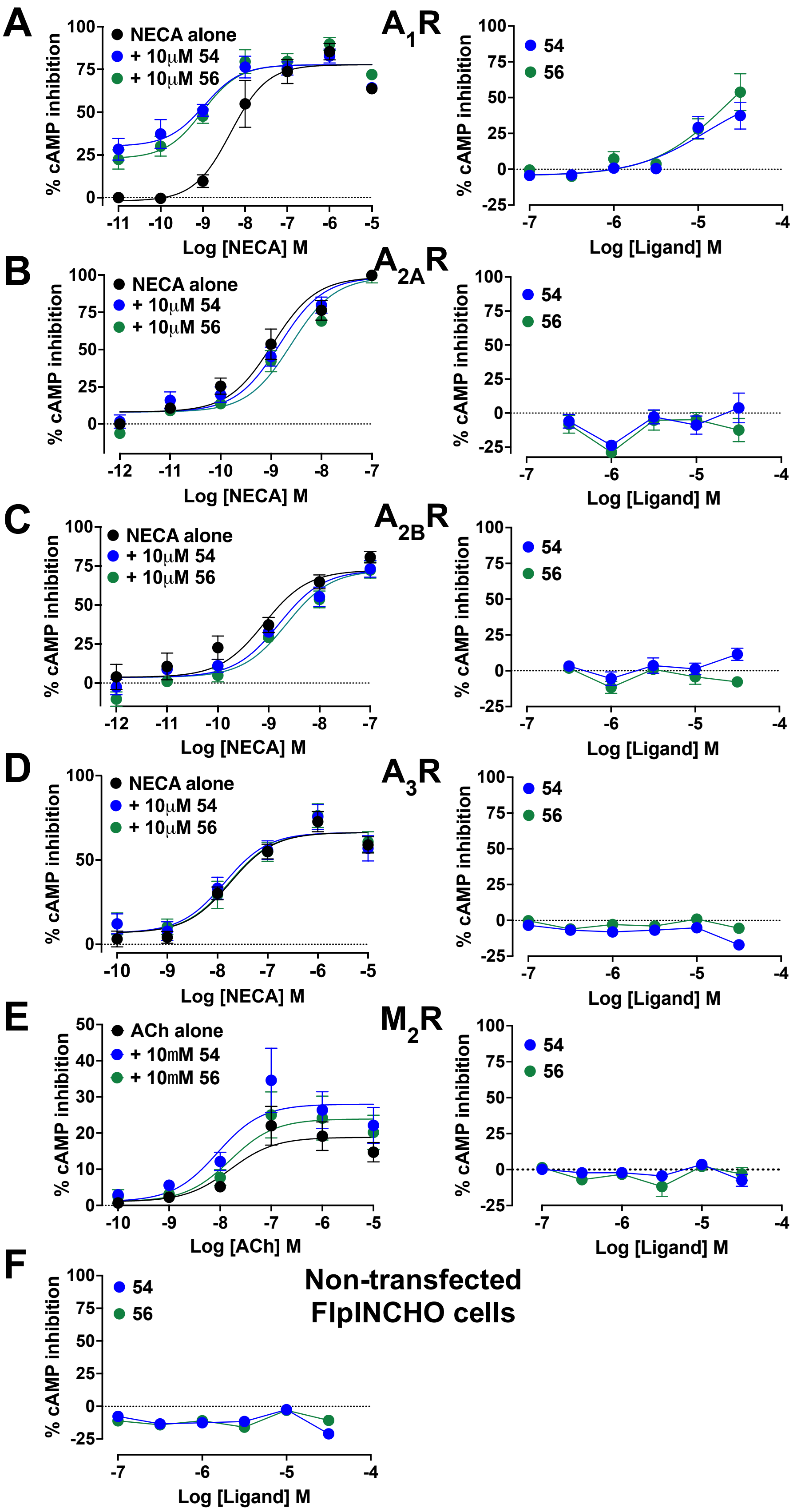
A
A$_1$R
NECA alone
+ 10μM 54
+ 10μM 56
% cAMP inhibition
Log [NECA] M
54
56
Log [Ligand] M
B
A$_{2A}$R
NECA alone
+ 10μM 54
+ 10μM 56
% cAMP inhibition
Log [NECA] M
54
56
Log [Ligand] M
C
A$_{2B}$R
NECA alone
+ 10μM 54
+ 10μM 56
% cAMP inhibition
Log [NECA] M
54
56
Log [Ligand] M
D
A$_3$R
NECA alone
+ 10μM 54
+ 10μM 56
% cAMP inhibition
Log [NECA] M
54
56
Log [Ligand] M
E
M$_2$R
ACh alone
+ 10mM 54
+ 10mM 56
% cAMP inhibition
Log [ACh] M
54
56
Log [Ligand] M
F
54
56
Non-transfected
FlpINCHO cells
% cAMP inhibition
Log [Ligand] M

Fig. S10. Selectivity of new A_1R PAMs **54** and **56** for enhancer and agonist activity. Allosteric modulation of orthosteric agonist (*left*) and allosteric agonism (*right*) of **54** and **56** were assessed using cAMP accumulation in A_1R (*A*), $A_{2A}R$ (*B*), $A_{2B}R$ (*C*), A_3R (*D*), M_2 receptor (*E*) and non-transfected (*F*) FlpINCHO cell lines. Data represent the mean ± SEM from n=3-8 individual replicates performed in duplicate. Error bars not shown lie within the dimensions of the symbol.
